# Oppositely Charged Homopolypeptides Form Ordered Helix Assemblies With Hierarchical Structure

**DOI:** 10.1002/smll.75495

**Published:** 2026-09-11

**Authors:** Ekaterina Buvalaia, Martin Dulle, Benoit David, Karoline Rengel, Michael Ohl, Beate Förster, Margarita Kruteva, Henrike Heise, Ralf Biehl, Holger Gohlke, Stephan Förster

**Affiliations:** ^1^ JCNS‐1 Forschungszentrum Jülich Jülich Germany; ^2^ IBG‐4 Forschungszentrum Jülich Jülich Germany; ^3^ ERC‐1 Forschungszentrum Jülich Jülich Germany; ^4^ Institute For Physical Chemistry Heinrich Heine University Düsseldorf Düsseldorf Germany; ^5^ IBI‐7 Forschungszentrum Jülich Jülich Germany; ^6^ Institute For Pharmaceutical and Medicinal Chemistry Heinrich Heine University Düsseldorf Düsseldorf Germany; ^7^ Institute of Physical Chemistry RWTH Aachen University Aachen Germany

**Keywords:** collagen, helix formation, polyarginine, polyelectrolyte complexes

## Abstract

Oppositely charged polyelectrolytes are known to form polyelectrolyte complexes. Polyelectrolyte complexes are typically structurally disordered. In recent years, complexes of oppositely charged polypeptides have received increasing attention. Polypeptides provide additional interaction motifs such as hydrogen bonds and salt bridges that could stabilize ordered assemblies. Under specific conditions, the formation of secondary structures has been reported. So far, it has been difficult to obtain long‐range ordered assemblies of polypeptide complexes. Here we show that liquid‐solid phase separation of oppositely charged homopolypeptides spontaneously leads to the formation of extended polypeptide helices that form highly ordered hexagonally or square‐packed helix arrays with helix–helix distances of 1.6–2.0 nm. The helices exhibit an additional periodic staggering along their axis with a periodicity of 15 – 30 nm, equal to the length of the extended helices. This hierarchical helix assembly was demonstrated for polyarginine assembled with polyaspartic acid or polyglutamic acid as examples. The materials can be shear‐aligned to achieve macroscopic uniaxial orientation of the helices. Crosslinking leads to solid elastic materials. Upon heating to temperatures above 40°C, the structure disorders due to a reversible helix‐coil transition. The structural and mechanical properties suggest a new route to synthetic peptide‐based materials.

## Graphical abstract

Hexagaonal and square assembly of polyarginine alfa‐helices formed in highly ordered polyarginine/polyaspartic acid complexes. The helices exhibit additional periodic staggering along their helical axis.

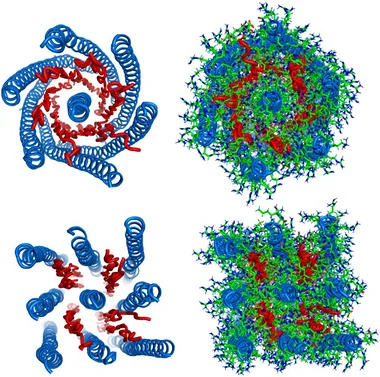

## 1 Introduction

Polyelectrolyte complexes are formed by associative interactions of oppositely charged polyanions and polycations, driven by electrostatic attraction and the accompanying release of counterions. These complexes are used in many applications, including separation membranes, surface coatings, ion‐exchange membranes, and drug delivery systems. Upon mixing, the polyelectrolytes undergo either a liquid/solid phase separation to form disordered solid complexes, or liquid/liquid phase separation into disordered gel‐like phases, which is currently intensively investigated, particularly for proteins [1]. In either case, complexes with proteins have received increasing attention in recent years with potential applications in biomedical engineering, drug delivery and synthetic biology [2, 3]. In principle, they offer the possibility to form complexes with defined secondary protein structures. As an example of a structurally simple system, polyarginine, which is known to form α‐helices, has already been investigated in forming complexes with polyanions [4], albeit in the disordered state. Also, the complexation of other polyamino acids with oppositely charged synthetic polyelectrolytes has been studied, with observations of liquid‐liquid or liquid‐solid phase separation, depending on charge density and ionic strength [5, 6, 7]. Perry et al. were the first that showed that solid phases with defined β‐sheet secondary structures can be obtained for pairs of chiral cationic and anionic polyaminoacids [8].

This indicates the possibility to assemble oppositely charged polypeptides into phases with defined secondary or possibly even higher‐order structures. This would be particularly attractive for the design of synthetic biomaterials. We therefore investigated the liquid‐solid and liquid‐liquid phase separation of polyarginine with polyaspartic acid and polyglutamic acid as a potential route to form charge‐stabilized helical assemblies. We find that indeed liquid/solid phase separation leads to the formation of stable α‐helices, which further assemble into highly ordered hexagonal or square arrays. Along the helices we find axial staggering with a periodicity that is equal to the length of the extended helices. The polypeptide complexes can be macroscopically aligned into uniformly oriented samples and crosslinked to obtain mechanically stable elastic materials.

## 2 Materials and Methods

### 2.1 Materials

Polyaspartic acid (PAsp) (Mw = 11 500 Da), polyglutamic acid (PGlu) (Mw = 13 000 Da) and polyarginine (PArg) (Mw = 19 000 Da) were obtained from Alamanda Polymers. Sodium chloride (NaCl ≥ 99.5%, Mw = 5844 g/mol) was obtained from Sigma–Aldrich. The chemicals were used without additional purification. Stock solutions of each polyaminoacid were prepared in deionized Milli‐Q water and were adjusted to have pH 7 using 0.1 m NaOH or 0.1 m HCl solutions. Impurities were removed by a 0.1 µm pore Anotop 10 size exclusion filter.

### 2.2 Complex Preparation

Polyelectrolyte complexes (PECs) were prepared in three steps: First, a NaCl solution with the desired NaCl concentration after mixing was prepared. Then PAsp or PGlu from the filtered stock solution was added, and the solution was vigorously vortexed for 15 s. Finally, the aqueous PArg solution was added, and the sample was also well mixed. Complexation occurred immediately after adding the polycation at the last step. After preparation, the samples were stored at 6°C.

### 2.3 Centrifugation

Centrifugation resulted in the formation of a white precipitate at low and high salt concentrations and a highly viscous liquid coacervate phase at medium salt concentrations. Centrifugation was done at 5236 g for 10 min at 21°C. The same properties were discovered at all polyaminoacid concentrations, which only changed the amount of the precipitate.

### 2.4 Optical Microscopy

The liquid‐liquid and liquid‐solid phase separation of complexes of PArg and PAsp was studied using optical microscopy (Zeiss Axiovert S100). After adding PArg at the last step of PECs preparation, the samples were put between two glass slides.

### 2.5 NMR

To investigate which and how much of the polyaminoacids form the solid precipitates, NMR experiments were performed (see Figure S24). Therefore, after mixing and centrifugation the polyaminoacids and supernatants were measured separately. For both PAsp/PArg and PGlu/PArg samples, we observe that at 0 mm NaCl a part of the negatively charged polyaminoacids stayed in the supernatant, i.e., the precipitate was formed mainly by PArg. For 2400 mm salt concentration, almost all PAsp/PGlu were in the supernatant, which could be observed by the precipitation of the compound of PAsp with oppositely charged ions from NaCl salt. It was already observed that PAsp tends to form hexagonal structures in wet powders ^4^.

### 2.6 ^13^C Solid‐State NMR

For solid‐state NMR measurements, ca 32 µg of PArg_100_/PAsp_100_ precipitate were packed into a 3.2 mm ZrO_2_ MAS rotor for solid‐State NMR spectroscopy. The sample was prepared by combining stock solutions of PArg_100_ and PAsp_100_, centrifuging, removing the supernatant, and freezing the precipitate. The precipitate was kept below 8°C until it was packed into the rotor. A ^13^C CP/MAS spectrum was recorded with a spinning speed of 11 kHz on a Bruker Avance III widebore NMR spectrometer operating at a field strength of 18.8 T (corresponding to 800 MHz ^1^H Larmor frequency). The sample temperature was set to ∼278 K by cooling the VT gas to a temperature of 273 K. The rf power for ^1^H 90° pulse and decoupling was 83 kHz, the contact time for cross polarization was 800 µs. For the spectrum, 512 scans with a delay time of 2 s were added. The FID was apodized with an exponential decay function with a line broadening of 50 Hz, and the spectrum was referenced to DSS using Adamantane as external standard.

### 2.7 Small‐Angle X‐Ray Scattering (SAXS)

To study the structure of the complexes, small‐angle x‐ray scattering (SAXS) experiments on the in‐house GANESHA instrument were performed. The x‐ray source of this laboratory‐based system was a D2‐MetalJet (Excillum) with a liquid‐metal anode operating at 70 kV and 3.57 mA with Ga‐Kα radiation (λ = 0.1341 nm), providing a brilliant and small beam (<100 µm). The beam was focused with a focal length of 55 cm using a specially made x‐ray optic (Xenocs) onto the sample position. Measurements were done in 2.1 mm boronsilicate glass capillaries (Hilgenberg, code 1409364, Germany). The transmitted intensity data were recorded by a position‐sensitive detector (PILATUS 300 K, Dectris). To cover the range of scattering vectors between 0.15 and 22 nm^−1^, different detector positions were used. The circularly averaged data were normalized to incident beam, counts per solid angle and measurement time before subtraction of the empty capillary and scaling to sample thickness afterward. All measurements were put on an absolute scale by standard‐less absolute intensity calibration.

### 2.8 Shear‐Alignment

Complexes of PArg_100_/PAsp_100_ and PArg_200_/PAsp_100_ prepared without added salt were shear‐aligned in an in‐house fabricated x‐ray transparent capillary shear cell, consisting of a rotating thin glass rod in a thin glass capillary. Shear‐alignment was performed at 60°C, just above the melting temperature of the complexes. After cooling back to room temperature, a heating‐cooling cycle of 10°C steps without further shearing was performed. At each temperature, the scattering patterns of shear‐aligned complexes were recorded.

### 2.9 Cross‐Linking

To preserve the ordered structure during drying, the polyaminoacid complexes were covalently cross‐linked. This was achieved by incubating the complex in a 0.6% aqueous glutardialdehyde solution for 24 h [9]. The crosslinked complexes were then rinsed with deionized water.

### 2.10 Stress–Strain Experiments

The stress–strain experiments on crosslinked polypeptide complexes were performed with a Linkam MFS stage.

### 2.11 Scanning Transmission Electron Microscopy (STEM)

S(T)EM was performed using a field emission Scanning Electron Microscope (SEM) (Volumescope, Thermo Fisher Scientific) equipped with a STEM detector operated at an acceleration voltage of 30 kV. Ultra‐thin sections were obtained by using a Leica Ultramicrotome UC7 cutting the complexes with a diamond knife (Diatome) at room temperature.

### 2.12 Differential Scanning Calorimetry (DSC)

DSC measurements were performed on a DSC Q2000 (TA Instruments) with a scan rate of 10 K/min.

### 2.13 Structural Modeling

The 3D structures of PArg/PAsp and PArg/PGlu hexagonal and square assemblies were modeled in two steps. Based on SAXS diffraction data, the Cα atom coordinates of each peptide chain were calculated using an in‐house Python script. This Cα model was used as a template to build the rest of the structure with MODELLER (version 10.4) [10]. To ensure the folding of arginine chains into α‐helices, distance restraints of 2.8 Ǻ were enforced between backbone carbonyl oxygen and amide nitrogen atoms of residues at positions *i* and *i*+4 during the modeling. The ROSETTA [11, 12] fast‐relax algorithm using the ref2015 [13] scoring function (version 2022.46) was used to optimize side chain packing. Considering that the length of a PAsp_100_ (or PGlu_100_) fully extended coil equals twice the size of a single PArg_100_ α‐helix, a single PAsp_100_ (or PGlu_100_) extended coil should be stabilized by pairs of PArg_100_ α‐helices placed consecutively (head‐to‐tail) along the entire coil length (i.e. 2 x PArg_100_ per PAsp_100_ (or PGlu_100_). To increase the performance of MD simulations, models including PAsp (or PGlu) random coils were truncated to half their size, i.e. one PArg_100_ per PAsp_50_ (or PGlu_50_). (Figure S14). A total of six such models were generated for subsequent MD simulations, including four hexagonal models (two in which PAsp/PGlu were considered fully α‐helical, and two in which PAsp/PGlu were modeled as extended coils), and two square models (in which PAsp/PGlu were modeled as extended coils).

### 2.14 MD Simulations

Steepest descent/conjugate gradient minimization (30 000 steps) was performed on these final models to optimize their geometry. Thermalization (Langevin thermostat [14], 300 K, 1 ns) and density equilibration (isotropic barostat at 1 atm., 300 K, 10 ns) were performed with the AMBER software suite [15] (version 24) using periodic boundary conditions with a time step of 1 fs. Positional restraints of 10 kcal mol^−1^ Ǻ^−2^ were applied during both thermalization and density equilibration steps. Classical MD simulations (8 replicas) were run with pmemd.cuda [16] for 200–500 ns in the NPT ensemble using a time step of 2 fs with the SHAKE algorithm [17]. The RESP charges [18] of the alkylated (*N*‐hexyl cap) C‐terminal residues were performed at the HF/6‐31G^*^ level of theory using Gaussian (version 09) [19] and their non‐bonded force field parameters were derived using the gaff force field [20] via parmchk2 from AmberTools 24 [15]. The simulation was performed using the ff14SB [21] force field and the TIP3P [22] water model. Long‐range electrostatic interactions beyond 9 Å were treated using the Particle Mesh Ewald algorithm [23]. The net positive charge of the system was neutralized by the addition of chloride ions using the tleap module. Solvation of the square systems was performed using PACKMOL [24] using a concentration of 3 m sodium chloride. MD snapshots were saved every 100 ps. Analyses were performed using the CPPTRAJ software [25] (AmberTools 24) [15]. In agreement with the radial distribution of guanidinium and carboxylate carbon‐carbon distances calculated from MD simulations (Figure S15) and with former computational studies, a cutoff distance of 4.5 Å between the carbon atoms of the guanidinium moiety of arginines and the carboxylate moiety of acidic residues was used to probe salt bridge interactions between PArg and PAsp (or PGlu) chains. The determination of the amount of α‐helical structures during MD simulations was performed using DSSP [26, 27].

## 3 Results and Discussion

To form ordered polypeptide complexes via liquid‐liquid or liquid‐solid phase separation it is advantageous to consider polypeptides that readily form stable secondary structures such as α‐helices or β‐sheets. We chose polyarginine as a suitable polypeptide, because it is known that polyarginine can undergo a coil‐helix transition in the presence of certain ions. Polyarginine has a guanidino group which in the presence of water is protonated to form a guanidinium cation. Due to the high cationic charge, polyarginine has a coil conformation in aqueous solution. If the cationic charges are screened, e.g. upon deprotonation at pH 10, by addition of > 1 m NaCl, or when anions with high binding affinity, such as surfactants, lipids or negatively charged polyelectrolytes, are added, it undergoes a coil‐helix transition. A particularly strong binding occurs for carboxylic acid groups, because of the stable guanidine‐carboxylic acid motif with double H‐bonding and electron delocalization. Therefore, polyarginine complexes with polyaspartic or polyglutamic acid appeared as promising candidates for ordered polypeptide helix assemblies.

## 4 Liquid‐Liquid and Liquid‐Solid Phase Separation

We investigated polyaminoacid complexes consisting of polyarginine (PArg) of different molecular weights or number of amino acids (aa) together with polyaspartic acid (PAsp) and polyglutamic acid (PGlu) with a number of amino acids of 100 aa. Table 1 and Figure S1 provide an overview of the investigated polypeptides.

**TABLE 1:** Average molecular weights and number of amino acids per chain of the polypeptides investigated in the present study. The molecular weights are given for the respective hydrochlorides. For all samples the polydispersities (PDI) are < 1.2 according to the supplier.

	Average Molecular Weight [g/mol]	Average number of repeat units [aa]
PArg_30_	5800	30
PArg_50_	9600	50
PArg_100_	19000	100
PArg_200_	38500	200
PAsp_100_	11500	100
PGlu_100_	13000	100
PSSH	17500	95

When mixing aqueous solutions of polyarginine (PArg) and polyaspartic acid (PAsp) or polyglutamic acid (PGlu), the oppositely charged polyaminoacids spontaneously phase separate to form complexes. Depending on temperature and the concentration of added salt (NaCl) we find either the formation of solid or liquid‐like complexes. A first identification of solid precipitates formed by liquid/solid phase separation and of liquid coacervates formed by liquid/liquid phase separation can be made using optical microscopy (Figure S1). Liquid complexes or coacervates appear as small, spherical fluid droplets, while solid precipitates appear as µm‐sized solid particles.

At room temperature, we observe a liquid‐solid phase separation with the formation of solid complexes at low salt concentrations c_s_ < c^*^ = 0.4 m and at high salt concentrations c_s_ > c^**^ = 2.4 m. For intermediate concentrations c^*^ < c_s_ < c^**^ we observe liquid‐liquid phase separation with the formation of microdroplets, as shown in Figure S1 for 0.8 m NaCl. In Figure 1 we show a phase diagram summarizing the observed stability regions of solid ordered complexes and of liquid disordered viscous or gel‐like complexes. Ordered solid complexes are preferably formed at high or low salt concentrations and lower temperatures. The structure of the ordered solids is determined by small‐angle x‐ray scattering as described in the next section.

**FIGURE 1:**
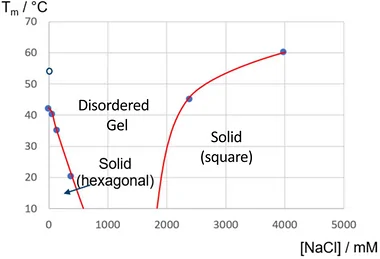
Phase diagram of the stability regions of ordered and disordered PArg/PAsp complexes as a function of salt concentration and temperature. The complexes are formed by liquid/solid‐phase separation at high and low salt concentrations, and by liquid/liquid phase separation at intermediate salt concentrations. The solid complexes show hexagonal or square ordering of PArg‐helices. Filled symbols denote melting temperatures of complexes with PArg‐chains consisting of 100 amino acids, open symbols denote complexes with PArg chains with 200 amino acids. The red lines are guides to the eye, taking into account that disordered gels are observed for salt concentrations of 800 and 1200 mm.

## 5 Nano and Helical Structure of Polypeptide Complexes

The structures of the PArg/PAsp and PArg/PGlu complexes prepared at different salt concentrations were investigated with small‐angle x‐ray scattering (SAXS). Figure 2 shows the measured SAXS‐curves for a 1:1 molar ratio of PArg_100_ and PAsp_100_ prepared at 2 mm total polyaminoacid concentration. We investigated complexes prepared at eight different added salt (NaCl) concentration ranging from *c_s_* =  0 mm (no added salt) to *c_s_* =  4 M. Except for *c_s_* =  800 and 1200 mm, which are in the liquid‐liquid phase separation regime c* < *c_s_* < c*, we observe a set of Bragg peaks in the intermediate *q*‐range of 3 < *q* < 10 nm^−1^ corresponding to the ordered assembly of helices upon liquid‐solid phase separation. At all salt concentrations there is a broad wide‐angle peak at q∼18 nm^−1^ corresponding to characteristic intra‐helix‐distances.

**FIGURE 2:**
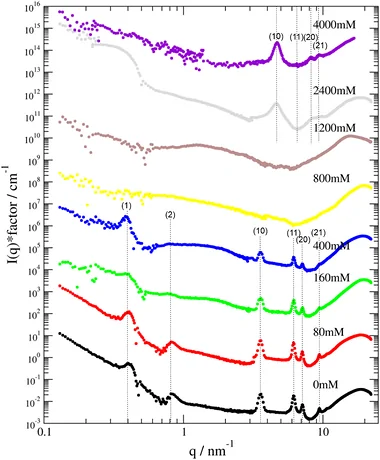
SAXS curves measured for 1:1 PArg_100_/PAsp_100_‐complexes prepared at 2 mm polymer concentration and different NaCl concentrations. The sets of indexed high‐*q* reflections observed for the solid complexes indicate the presence of highly ordered hexagonal or square arrangements of helices. The low‐*q* reflections indicate a lamellar periodicity. For intermediate salt concentrations (800, 1200 mm) we observe liquid complexes with no ordered arrangements.

In the low salt regime for *c_s_* =  0 – 400 mm we observe a set of Bragg peaks with *q*‐ratios $1:\sqrt{3}$:2:$\sqrt{7}$ with the *q*‐value of the primary peak at *q*  =  3.6 nm^−1^. This corresponds to a 2D‐hexagonal lattice, where the peak positions are given by

(1) $$q_{hk}=\frac{4\pi }{\sqrt{3}a}\;\sqrt{h^{2}+hk+k^{2}}$$

We interpret the observed peak positions to correspond to a long‐range ordered hexagonal packing of helices with helix‐helix distances or helix Bragg‐spacings of *a* = 2.0 nm (Figure 3a). We also observe a pronounced form factor minimum at *q*_10_ =  8 nm^−1^ corresponding to a cylinder diameter of 1.0 nm, likely related to the diameter of the helix backbone. In addition, we observe a low‐*q* peak at *q_d_* =  0.42 nm^−1^ with a second‐order peak at 0.84 nm^−1^, from which we calculate a characteristic repeat distance or long‐range Bragg‐spacing of $d=\frac{2\pi }{q_{d}}=$15 nm. This matches the length of an extended α‐helix well, which is *L*  = *N_aa_* *l*  =  15.0 nm with *N_aa_* =  100 and *l*  =  0.15 nm the axial distance between amino acids.

**FIGURE 3:**
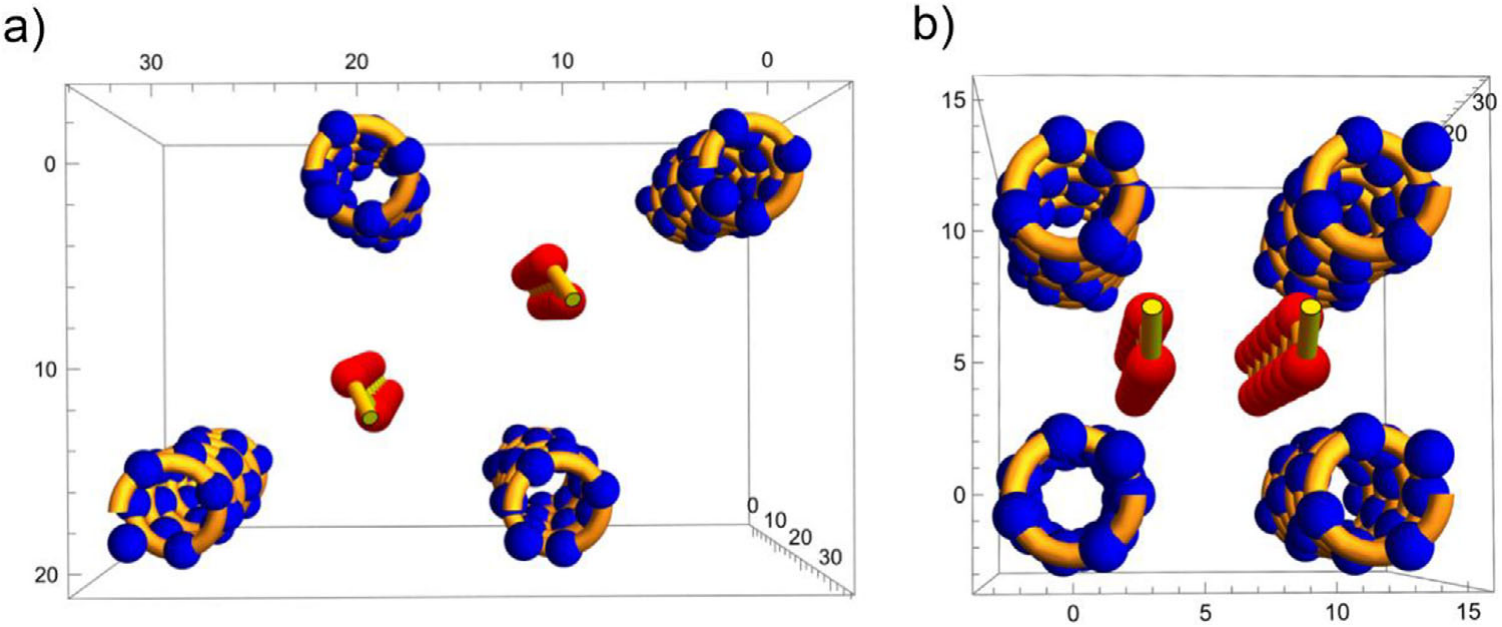
Idealized structures of the hexagonal (a) and square (b) unit cells with the backbones of the PArg‐helices (blue) and the PAsp‐ or PGlu‐chains (red) indicated. The dimensions are given in Ångströms. The longitudinal repeat distance of the red zig‐zag‐chain backbones is adjusted to fulfill 1:1 charge stochiometry per unit cell (see also Figure S13).

In the high‐salt regime for *c_s_* =  2400 – 4000 mm we observe a set of Bragg peaks with *q*‐ratios $1:\sqrt{2}$:2:$\sqrt{5}$ with the primary peak position at *q*  =  4.3 nm^−1^ corresponding to a long‐range ordered square packing of helices (Figure 3b). For this lattice the peak positions are given by

(2) $$q_{hk}=\frac{2\pi }{a}\;\sqrt{h^{2}+k^{2}}$$

with helix‐helix distances or helix Bragg‐spacings of *a* = 1.52 nm. We also observe a form factor minimum at *q*_10_ =  7 nm^−1^ corresponding to a helix diameter of 1.5 nm. For the high‐salt concentrations we do not observe any low‐*q* peaks.

We further investigated differences in the helical order when comparing different polyanions such as PAsp and PGlu (Figure 4 a/b), when using different polyamino acid chain lengths or number of amino acids (Figure 4 c/d), or different polycation:polyanion stoichiometries (Figure 4 e/f) using small‐angle x‐ray scattering.

**FIGURE 4:**
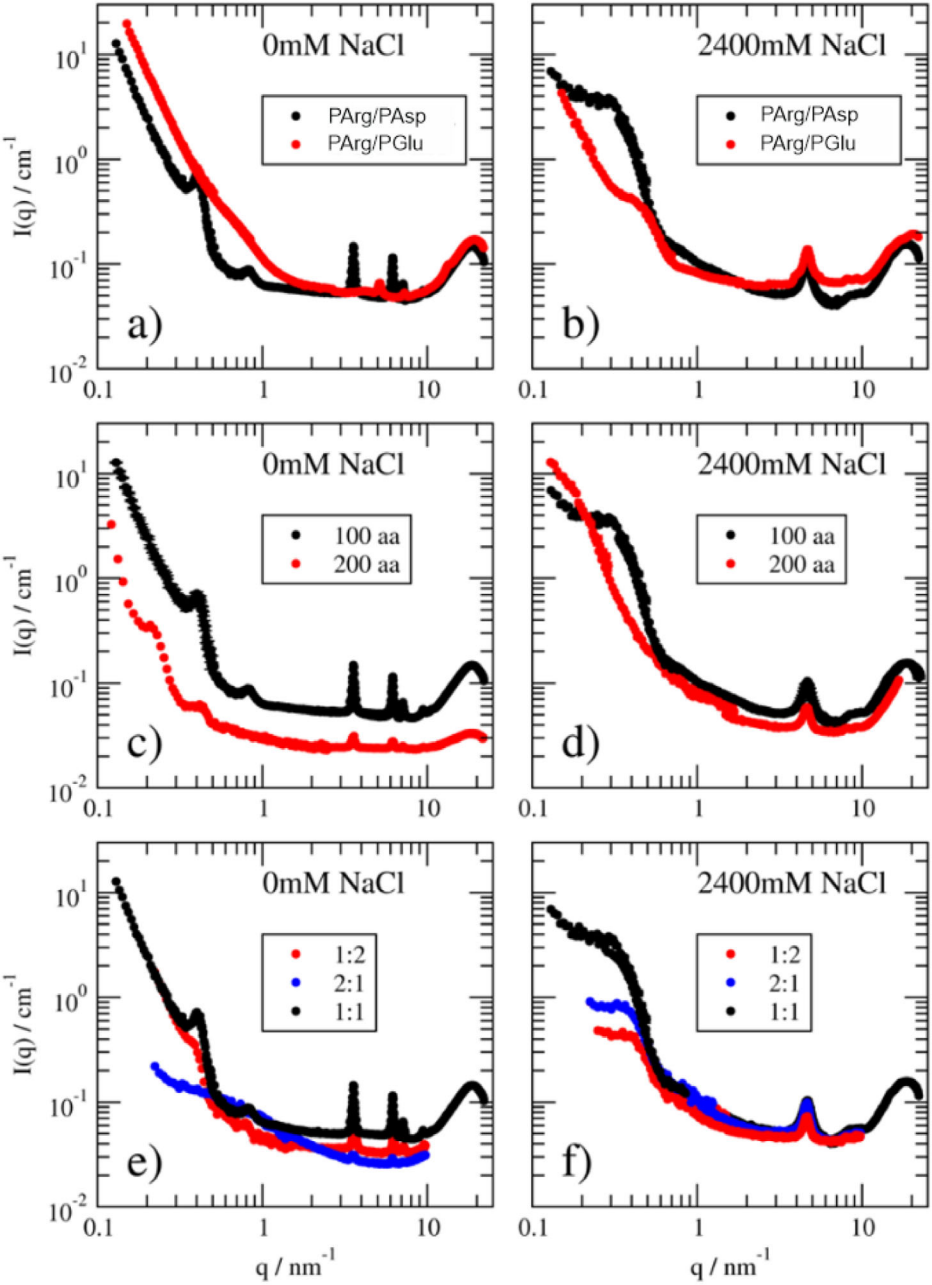
SAXS curves for PAsp_100_/PArg_100_ complexes prepared at 2 mm polyaminoacid concentration and two different NaCl concentrations in comparison to PGlu/PArg complexes (a, b), for different lengths or amino acid numbers (c, d), and for different stoichiometries of 1:2, 1:1, and 2:1 (e,f). Upon variation of the parameters, we see transitions from hexagonal to square arrangements of helices (a, b), shifts in the low‐q peak position (c, d), and changes in the scattering intensity at constant peak positions in (e, f).

Figures 4 a/b show the SAXS‐curves for PArg_100_/PGlu_100_‐complexes prepared at 0 and 2400 mm salt concentration, compared to the SAXS‐curves of the PArg_100_/PAsp_100_ complexes. For the PArg/PGlu complexes at both low salt (0 mm NaCl) and high added salt (2400 mm NaCl) we observe Bragg peaks with *q*‐ratios $1:\sqrt{2}:4$ indicating a square assembly of helices. With the first Bragg peak at *q*  =  5.2 nm^−1^ we calculate a helix‐helix distance or helix Bragg‐spacing of *a*  =  1.22 nm. This can be compared to PArg_100_/PAsp_100_‐complexes, which form hexagonal helical order at low salt concentrations and square helical order at high salt concentrations. The helix–helix distances are 1.52 nm for square packing, and thus slightly larger compared to the value of 1.22 nm for the PArg_100_/PGlu_100_‐complexes. We also investigated the complexation of PArg_100_ with polystyrene sulfonic acid (PSSH, 95 repeat units) at low and high salt concentrations, but did not observe any order in the SAXS‐curves.

We also investigated solid complexes of polyarginines of different chain lengths or number of amino acids (aa = 30, 50, 100, 200) with PAsp_100_ at stochiometric 1:1 charge ratios. PArg_200_/PAsp_100_ prepared at low‐salt (*c_s_* =  0 mm NaCl) and high‐salt (*c_s_* =  2400 mm NaCl) concentrations show the same peak positions at intermediate and high *q*, indicating the same helix packing when comparing to PArg_200_/PAsp_100_ as shown in Figure 4c,d. However, for PArg_200_/PAsp_100_ the low‐*q* peaks are shifted from *q_d_* =  0.42 to *q_d_* =  0.2 nm^−1^, with a second‐order peak at 0.4 nm^−1^. From the first peak position, we calculate a characteristic distance or long‐range Bragg‐spacing of $d=\frac{2\pi }{q_{d}}=$ of 31 nm in agreement with the expected doubling of the helix length, which was 15 nm for PAsp_100_. For PAsp_30_ and PAsp_50_ we did not observe any Bragg peaks indicating a disordered helical arrangement (Figure S8).

Furthermore, we investigated off‐stochiometric compositions of polycations and polyanions. For PArg_100_/PAsp_100_‐complexes we varied the ratio PArg:PAsp ratio from 1:2 to 1:1 to 2:1. This did not affect the peak positions as seen in Figure 4 e/f. For the 1:1 ratio we observed the highest scattering intensity. This indicates a preference for 1:1 charge compensation or ion binding of polycations and polyanions. It is known that there is a particularly strong ionic binding between arginine and aspartate residues [28, 29]. Also, changes of the overall polyamino acid concentrations or different centrifugation conditions did not affect the structure, as is shown in the SI. ^1^H‐NMR spectra of the supernatants (Figure S22) show that at low salt concentrations some PAsp‐chains remain in the supernatant. At high salt concentration, nearly all PAsp‐chains remain in the supernatant, indicating that ordered complex formation is largely dominated by PArg‐salt interactions. This explains that the measured SAXS‐curves at high salt concentrations (Figures 2 and 3, Figures S2–S7) all show square helix assemblies with the same structure, irrespective of the salt and polymer concentration, or the type of polyanions (PAsp, PGlu).

For Arg_100_/Asp_100_ complexes we performed ^13^C MAS solid‐state NMR to further assess the conformation of the polyaminoacid chains in the solid complex. As shown in Figure S28 we observe a sharp C_α_‐signal at 60 ppm, which has a positive secondary chemical shift of 3 ppm, indicative of α‐helical conformation and high conformational homogeneity.

For PArg_200_/PAsp_100_ we additionally investigated the structure of the ordered complexes prepared at no added salt by scanning transmission electron microscopy (STEM). Ultra‐thin sections of the samples were prepared by ultramicrotomy. The STEM‐image in Figure 5 shows a diagonal stripe pattern, corresponding to a projection of the side view of the helix assembly. The diameter of the stripes is ∼7 nm, which is larger than the unit cell dimension *a*, indicating a projection along an oblique direction with respect to the helical axis, or a weak modulation of the helical order orthogonal to the helical axis.

**FIGURE 5:**
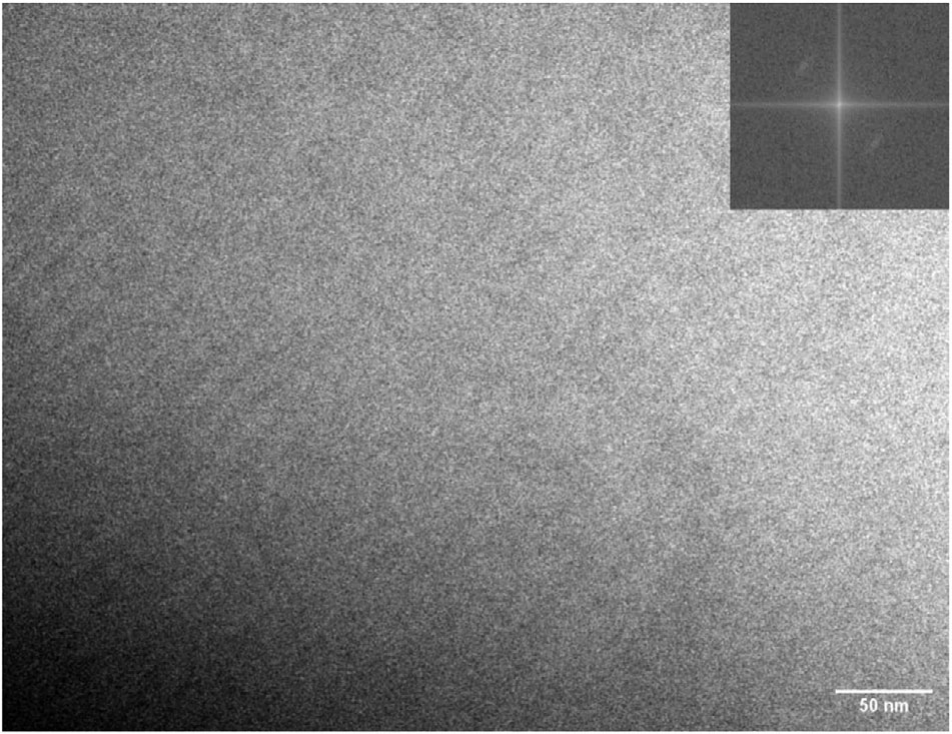
S(T)EM‐image obtained at an acceleration voltage of 30 kV of ultramicrotomed ultra‐thin sections of PArg_200_/PAsp_100_ complexes prepared at no added salt. Despite the weak contrast of the unstained sample we observe a stripe pattern along the diagonal, corresponding to a projection of the side view of the helix assembly. The stripes have diameters of ∼ 7 nm.

Therefore, we find that PArg/PAsp and PArg/PGlu complexes at low salt concentrations form solid materials consisting of stable extended helices that assemble into long‐range ordered hexagonal or square arrangements with additional long‐range periodicity along their helices. This is remarkable, as polyelectrolyte complexes usually do not show any order. As an exception, we note one report of SAXS measurements on complex coacervate micelles of polylysine‐PEG block copolymers with dsDNA [30], which show a single Bragg peak at *q_d_* =  2.2 nm^−1^, which corresponds to an ordered structure with a periodicity of 2.9 nm.

## 6 Uniaxial Orientation

Since the polyaminoacid complexes show helical and fiber‐like order, we investigated whether the helices in the PArg/PAsp complexes could be shear aligned. For this, PArg_100_/PAsp_100_ and PArg_200_/PAsp_100_ prepared without added salt were shear‐aligned in a capillary shear cell. Shear‐alignment was performed by heating above the melting temperature to 60°C, and cooling back during shear to room temperature in 10°C steps. The scattering patterns of shear‐aligned PArg_100_/PAsp_100_ complexes are shown in Figure 6.

**FIGURE 6:**
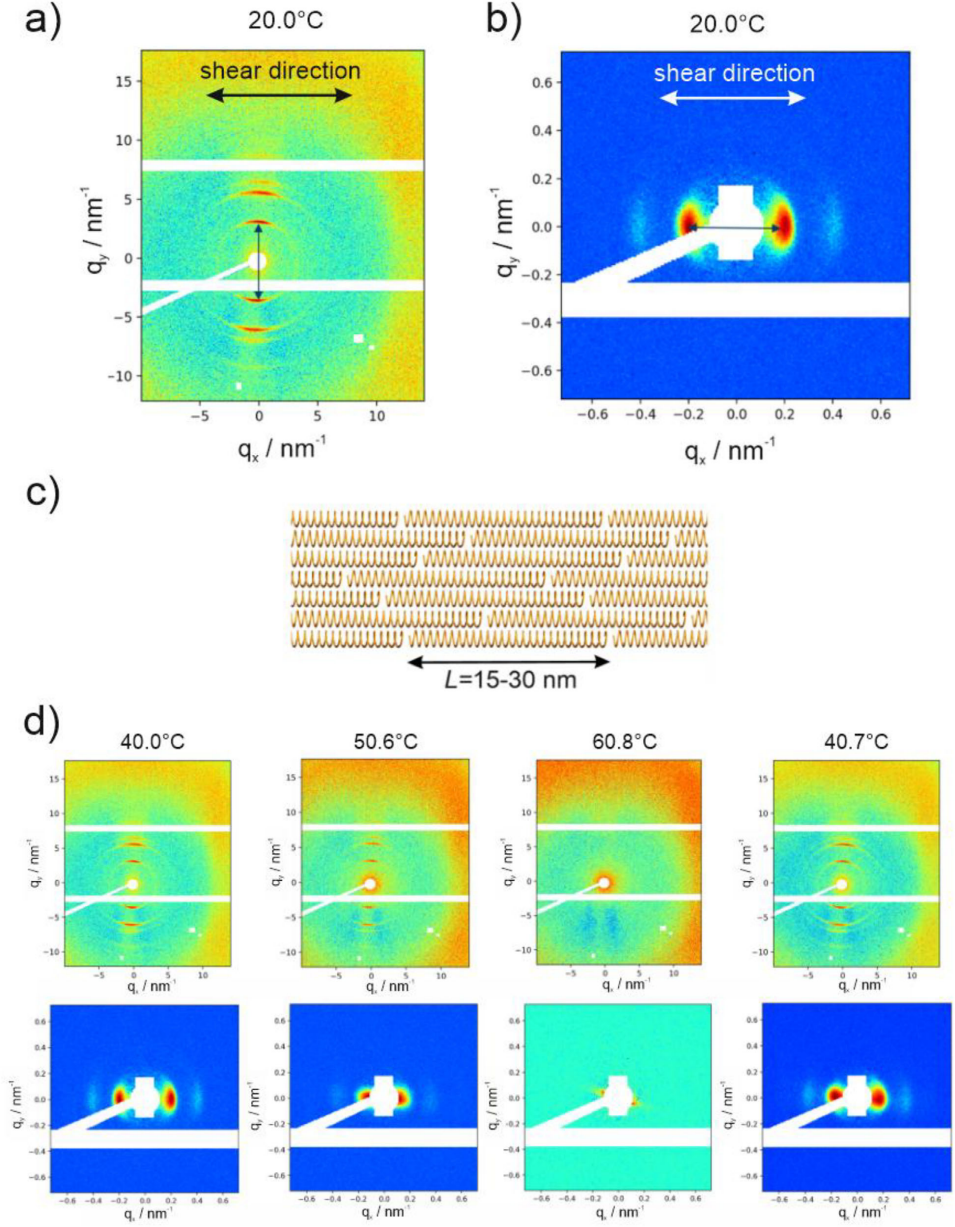
Measured SAXS patterns for shear‐oriented PArg_200_/PAsp_100_ complexes prepared at 2 mm polyaminoacid concentration with no added salt. a) SAXS pattern in the intermediate *q‐*range (top) and b) SAXS‐pattern in the low *q‐*range for complexes that were shear‐oriented in the horizontal direction. We observe sharp Bragg peaks at intermediate *q* along the meridian, corresponding to an oriented hexagonal helix arrangement with the helical axis parallel to the shear direction (c). The low‐*q* Bragg peaks on the equator indicate an additional periodicity along the helix axis with a long period L corresponding to the extended helices. d) SAXS‐patterns during a heating/cooling cycle. All reflections disappear when heating to 60°C, above the melting temperature of *T_m_* = 53°C. The peaks reappear upon cooling.

In the SAXS patterns shown in Figure 6, shear‐alignment was performed in the horizontal direction. The SAXS patterns exhibit the intermediate‐*q* peaks corresponding to the hexagonal helix arrangement as sharp arcs located on the meridian (Figure 6a). This indicates that the helices are aligned horizontally in the shear direction. In Figure 6b we show the scattering pattern obtained for a large sample‐detector position, where the low‐*q* peaks can be observed. They are located on the equator, indicating that the long‐range periodicity is parallel to the helical axis. The broad wide‐angle Debye–Scherrer ring at high *q* (q∼18 nm^−1^) corresponds to the intra‐helix structure. Alternatively, a smectic arrangement of helix layers, having in‐plane hexagonal ordering, could also be conceivable. However, shear in horizontal direction would align the smectic layers parallel to the shear direction, such that the low‐q peaks would be located on the meridian, and the intermediate‐*q* peaks on the equator, which is not experimentally observed.

In the literature we find one report of an x‐ray diffraction study on shear‐aligned PArg hydrochloride α‐helices. The respective diffraction patterns feature a set of hexagonal packed helix‐helix spacings of d = 1.25 – 1.37 nm, increasing with the hydration level [31]. Therefore, the α‐helical structure PAsp in the complexes is the same as for PArg hydrochloride, just the helix‐helix spacing is smaller, because of the lower molar volume of the hydrochloride.

## 7 Melting Transition

For the PArg‐complexes we observe a reversible melting of the ordered structure as shown in Figure 7 for PArg_100_/PAsp_100_ prepared at no added salt. When the sample is heated from 20°C to 42°C, the SAXS curves show a loss of both the low‐*q*‐ and high‐*q* peaks at *T_m_* = 41°C (Figure 7a). When the sample is heated to the molten state and cooled back down to 20°C (Figure 7b) we observe re‐ordering with the re‐appearance of both low‐*q*‐ and high‐*q* peaks. We thus observe a quite sharp, reversible melting transition, which we attribute to a helix‐coil transition.

**FIGURE 7:**
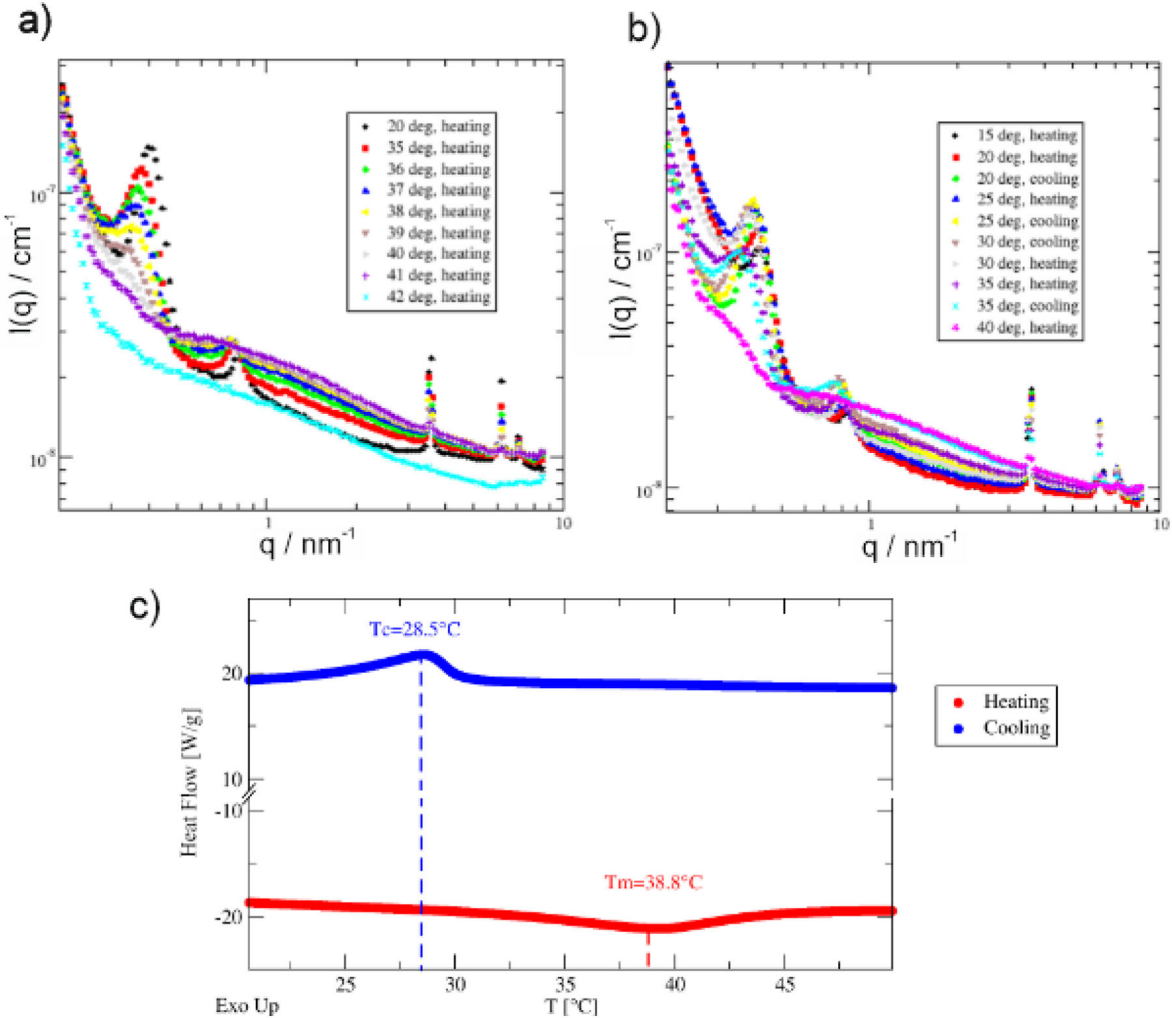
(a) SAXS‐curves of PArg_100_/PAsp_100_ complexes recorded during heating above the melting temperature of T_m_ = 41°C. (b) SAXS‐curves of PArg_100_/PAsp_100_ complexes recorded during a 15°C → 40°C → 20°C heating/cooling cycle to show the reversibility of the melting transition. The melting transition is indicated by the loss of both the low‐*q* and the high‐*q* Bragg peaks. (c) DSC‐trace during the heating/cooling cycle showing the phase transitions upon heating and cooling.

We investigated the melting transition also with differential scanning calorimetry (DSC). The DSC trace in Figure 7c shows a phase transition at 38.8°C upon heating, and a reverse transition at 28.5°C upon cooling. The observed hysteresis indicates a first‐order transition. The hysteresis observed in the DSC heating/cooling experiments is larger compared to the hysteresis in the SAXS heating/cooling experiments because of the much higher heating/cooling rates of 10 k/min in the DSC experiments. The measured transition enthalpies for the heating and cooling curves are in a range of ΔH = 2.2 – 2.6 J/g. Estimating a mass fraction of the PArg‐chains of ca. 50% (f_Arg_ = 0.5) and taking the molar mass of an arginine residue (M = 174 g/mol), we calculate a transition enthalpy per residue of $\Delta \;H_{r}=\frac{M\Delta H}{f}=0.8-0.9$ kJ/mol, which is smaller than the range of 1.7 – 3.8 kJ/mol reported for a range of proteins with uncharged amino acid residues [32]. This may indicate that the presence of repulsive charges along the helix significantly reduces the enthalpy of the PArg‐helix formation, or that the PArg helix conformation does not fully relax to a random coil conformation upon heating. An indication for the latter is the reappearance of oriented azimuthal reflections upon cooling, as described in the next section.

Figure 6d shows measured SAXS patterns of a shear‐oriented PArg_200_/PAsp_100_‐complex during a heating/cooling cycle. The full set of SAXS patterns are shown in (Figure S11). We observe that both the mid‐*q* reflections on the meridian and the low‐*q*‐reflections on the equator disappear when heating to 60°C, above the melting temperature of *T_m_* = 53°C. Surprisingly, both sets of peaks reappear at their previous positions upon cooling. We originally expected to observe Debye‐Scherrer rings upon cooling, related to randomly oriented helix directions during re‐crystallization. Obviously, the polyaminoacids remain in an extended oriented state after melting, such that the orientation is preserved upon recrystallization, at least over the duration of the heating/cooling experiment. This is a behavior not usually observed for polymer chains.

## 8 Crosslinking

The polyaminoacid complexes are malleable white solid materials and therefore interesting for biomedical applications. Freshly prepared complexes contain water, which slowly evaporates over time. Thereby, the complexes lose their ordered helix assembly structure. To preserve the ordered structure upon drying, the polyaminoacid helix assembly was covalently crosslinked. This was achieved by immersing the complex in an aqueous glutardialdehyde solution [9]. Using SAXS, we investigated the structural changes during drying of the cross‐linked material. Figure 8 shows that crosslinking preserves the helix arrangement upon drying. Interestingly, during drying we observe a reversible transition from a hexagonal to a square cylinder assembly. Thereby the characteristic hexagonal peak position ratios 1:$\;\sqrt{3}:2:\sqrt{7}$ change to the characteristic square peak position ratios $1:\sqrt{2}$:2:$\sqrt{5}$. The unit cell dimensions change from *a_hex_* =  2.0 nm to *a_sq_* =  1.66 nm. This corresponds to the reduction of the unit cell base area from $A_{hex}=\frac{\sqrt{3}}{2}\;\;a_{hex}^{2}=3.5$ nm^2^ to $A_{sq}=a_{sq}^{2}=2.8$ nm^2^. This indicates that the freshly prepared samples contained ca. 20% water. Upon re‐wetting the hexagonal arrangement is reformed. This demonstrates that crosslinking stabilizes the ordered helix arrangement, thus obtaining a stable solid material suitable for biomedical applications. The crosslink density seems to be high enough to stabilize the structure but low enough to still allow a transition between hexagonal and square structures.

**FIGURE 8:**
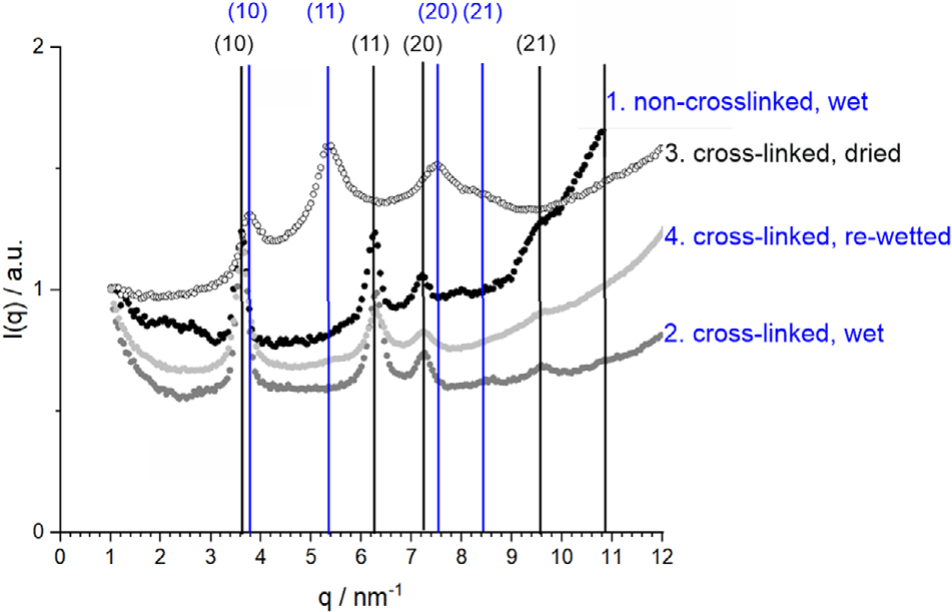
SAXS‐curves of covalently crosslinked PArg_100_/PAsp_100_ complexes in the crosslinked wet (2), dry (3), and re‐wetted state (4), together with the SAXS‐curve of a non‐crosslinked sample in the wet state as a reference (1). We observe that crosslinking preserves the ordered helix structure, with a reversible hexagonal – square transition upon drying. The black and blue vertical lines indicate the characteristic peak positions for the hexagonal (black) and square (blue) helix assemblies.

The mechanical properties of cross‐linked PArg_100_/PAsp_100_ complex were investigated by stress–strain experiments as shown in Figure 9. From the linear increase at strains < 5% we determine a tensile modulus of *E* = 6 MPa. Between strains of 5% and 30% we observe plastic deformation with a tensile strength of 0.3 MPa and an elongation at break of 20%.

**FIGURE 9:**
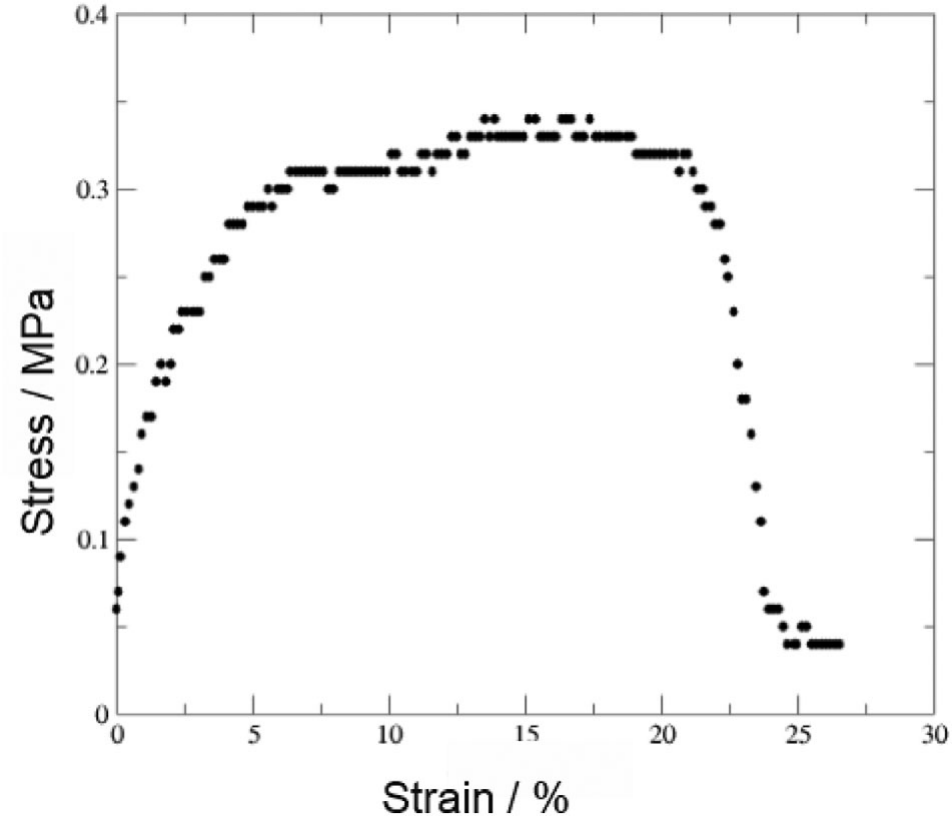
Stress–strain curve of a crosslinked PArg_100_/PAsp_100_ complex showing the elastic behavior and mechanical performance.

## 9 Helical Assembly Model

Based on the structure analysis by SAXS and considering local charge neutrality, we propose a model for the structure of the helical PArg/PAsp‐ or PArg/PGlu‐assemblies as shown in Figure 3, and also Figure S13. For an α‐helix we have a helix pitch of *d_p_* =  0.54 nm and a number of *n_aa_* =  3.6 amino acid residues per pitch. For PArg_100_ we therefore have an extended helix length of $L=\frac{100d_{p}}{n_{aa}}=15$ nm, in good agreement with the low‐q Bragg peak positions (Figures 2 and 5). The vertical distance per amino acid residue along the helix direction is $d_{z,aa}=\frac{d_{p}}{n_{aa}}=0.15$ nm. If all amino acid residues are charged, this corresponds to a charge density in the vertical direction of $q_{z}=\frac{1}{d_{z,aa}}=6.7$ e/nm. The contour length per amino acid of an extended polypeptide chain can vary between 0.34 and 0.38 nm, depending on the amino acid residue. We here assume a value of *d_aa_* =  0.37 nm. The hexagonal and square unit cells have one α‐helix per cell. If extended in the z‐direction, the PAsp or PGlu chains have a charge density of 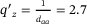 e/nm.

As at neutral pH PArg is in the protonated state and PAsp and PGlu are in the carboxylate states, local charge neutralization by attractive Coulomb forces is a strong binding force for complex formation. The guanidino group of the arginine residue is known to form stable ionic double bonds or salt bridges with carboxylic acids such as aspartic acid or glutamic acid. We therefore consider vertical charge compensation *q_z_* = *q*′_*z*_  as a strong requirement for packing in the unit cell. We therefore propose two PAsp or PGlu chains per unit cell, and have the chains in a zig‐zag conformation with an off‐vertical angle of $\gamma =\arccos(\frac{2d_{z,aa}}{d_{aa}})\;∼36$°. Each PAsp_100_ or PGlu_100_ chain then has an extended length of *L*  =  100 *d*_*z*,*aa*_ =  15 nm, i.e. the same extended length as the PArg_100_‐helix. In a hexagonal unit cell, the two chains can be placed at positions $\left(\frac{a}{2},\frac{a}{2\sqrt{3}}\right)$ and $\left(a,\frac{a}{\sqrt{3}}\right)$, where *a* is the unit cell size (see, Figure S13a, b). The PArg‐helices are located at the vertices of the unit cell. We observe reversible transitions from hexagonal to square unit cells. With small rearrangements of the PArg‐helices and PAsp or PGlu‐chains, a possible structural model for the square unit cell is to place the two PAsp or PGlu chains at positions $\left(\frac{a}{4},\frac{a}{2}\right)$ and $\left(\frac{3a}{4},\frac{a}{2}\right)$, and the PArg‐helices at the vertices of the unit cell (Figure S13c, d). At a square → hexagonal phase transition, the corresponding areas of the unit cells are

(3) $$A_{hex}=\frac{\sqrt{3}}{2}\;\;a_{hex}^{2}=A_{sq}=a_{sq}^{2}\;$$

It follows that the square → hexagonal transition involves an increase of the helix‐helix distance from $a_{sq}\to \;a_{hex}=\frac{2^{1/2}}{3^{1/4}}\;a_{sq}∼1.07a_{sq}$. This implies that a square→hexagonal phase transition is driven by increased helix‐helix repulsion, e.g. by hydration as shown in Figure 8. We note that the above structural considerations of the unit cell correspond to idealized structures of the polyaminoacid backbones and do not consider the spatial distribution of the amino acid residues which fill most of the unit cell space. If only the backbone positions were considered, the intensity of certain lower‐order Bragg peaks should be reduced. As this is not the case in the experimental SAXS‐curves, it indicates that the unit cell is rather homogeneously filled with amino acid residues of the PArg‐helices and the extended PAsp‐ and PGlu‐chains.

## 10 MD Simulations

MD simulations were performed (see Materials and Methods section) to probe the dynamics of PArg_100_/PAsp_50_ and PArg_100_/PAsp_50_ hexagonal and square assemblies at the atomistic level (Figure 10). The average structures of both the hexagonal and square assemblies computed from concatenated MD trajectories display a right‐handed superhelix, likely resulting from the omission of additional negatively charged chains at the interface between adjacent external PArg_100_ helices, which would normally be present in an extended crystal lattice (Figure S16). Yet, this intrinsic tendency to form right‐handed superhelices bears a notable parallel to the right‐handed superhelix or triple helix of collagen.

**FIGURE 10:**
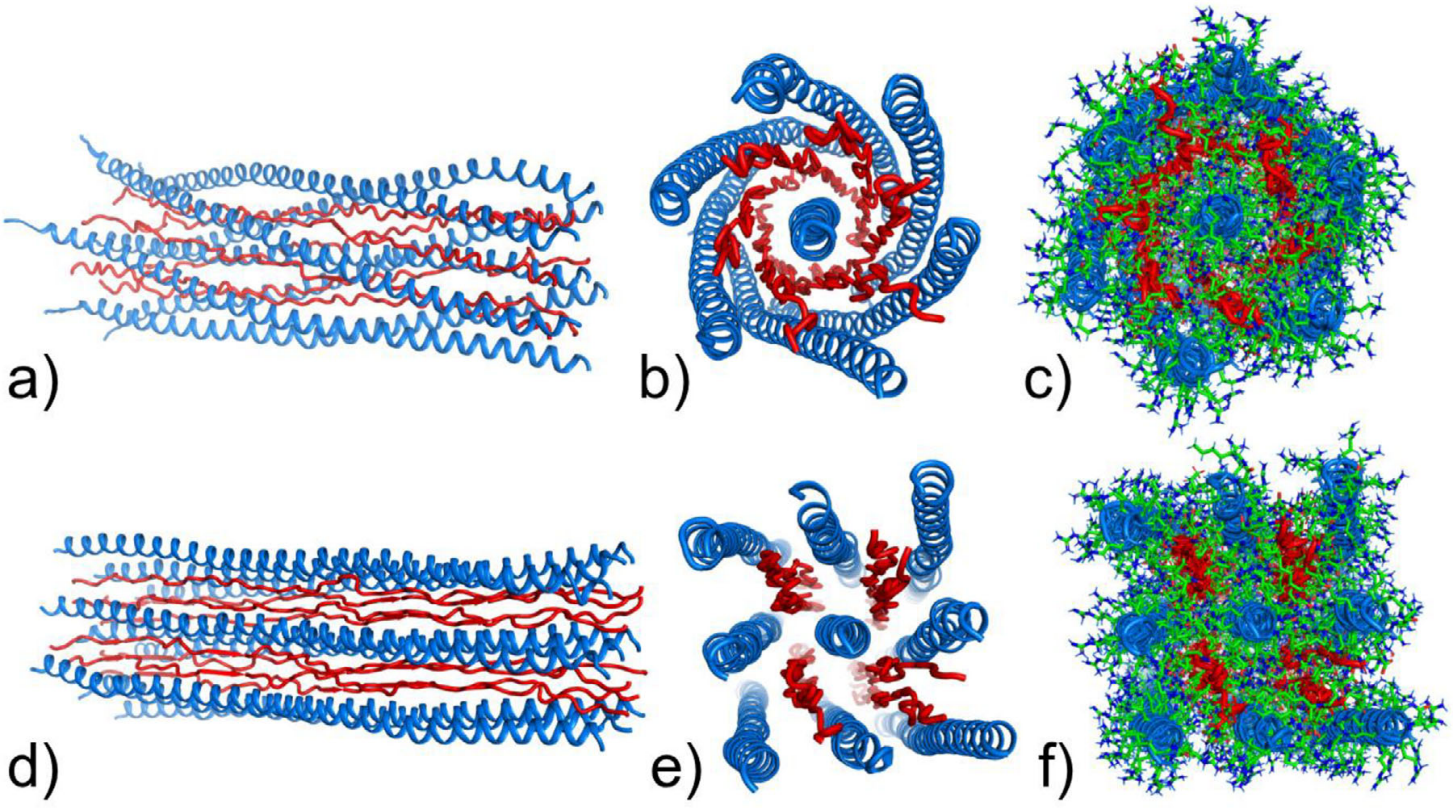
Average structures computed from concatenated MD trajectories. Hexagonal assembly: (a) backbone longitudinal view, (b) backbone transverse view, (c) space‐filling transverse view with all atoms visible (side chains in green). Square assembly: (d) backbone longitudinal view, (e) backbone transverse view, f) space‐filling transverse view. Blue helices: PArg_100_. Red coils: negatively charged polypeptides (PAsp_50_, PGlu_50_).

Despite rare and transient localized structural unfolding (Figures S17 and S18), PArg_100_ chains in both hexagonal and square assemblies preserve their overall α‐helicality, irrespective of the composition of the negatively charged polypeptides (PAsp or PGlu) they interact with (Figure 11). Notably, in agreement with experimental measurements (Figures 2 and 4), sampled distances between Cα atoms of equivalent residues from adjacent PArg_100_ chains in both square and hexagonal models follow a Gaussian distribution centered around 1.6 nm (square) and 2.0 nm (hexagonal), respectively (Figure 11a). While most Cα‐Cα distances in the hexagonal models adopt values around 2.0 nm, larger Cα‐Cα distances were observed between PArg_100_ N‐termini in the PArg_100_/PAsp_50_ model due to transient structural unfolding (Figure S19). In contrast, PArg_100_ N‐termini in the PArg_100_/PGlu_50_ hexagonal model (Figure S20) and in both square models (Figures S21 and S22) showed a lower structural variability. In square assemblies, higher Cα‐Cα distances beyond 1.6 nm may also stem from local repulsion of neighboring positive charges due to the increased compactness of the square assemblies in comparison to the hexagonal ones.

**FIGURE 11:**
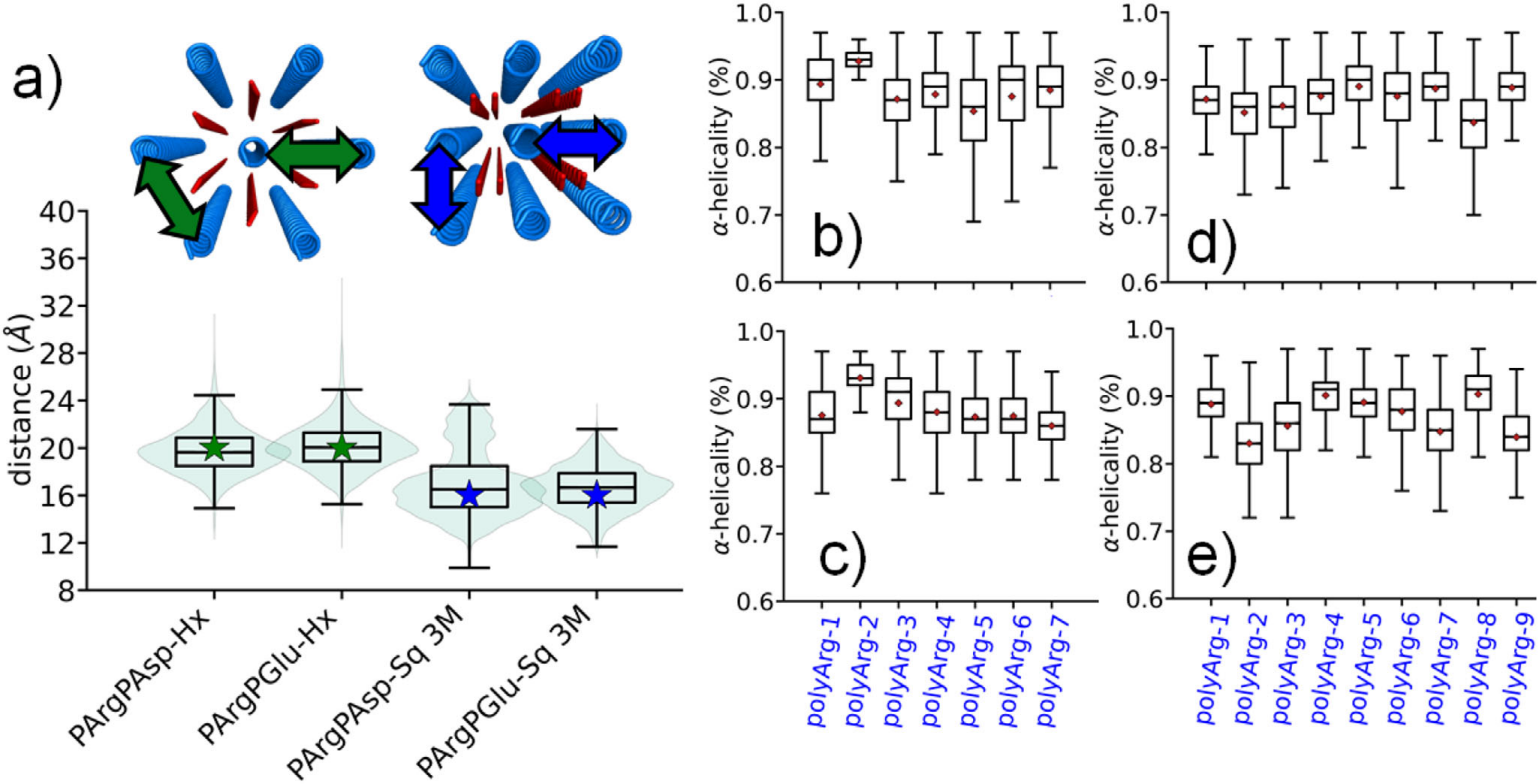
Stability of PArg_100_ helices. (a) Sampled distances between the Cα atoms of every tenth equivalent residue in adjacent α‐helices (excluding N‐termini) during MD simulations. Green star/arrow: ideal distance between adjacent α‐helices in the hexagonal model. Blue star/arrow: ideal distance between adjacent α‐helices in the square model. Cyan surface: corresponding kernel density estimations. Hx: hexagonal assemblies. Sq 3m: square assemblies simulated in 3 m NaCl. (b–e) α‐helical content of individual PArg_100_ chains determined by DSSP ^26,27^ for hexagonal PArg_100_/PAsp_50_ (b) and PArg_100_/PGlu_50_ (c) assemblies, and for the square PArg_100_/PAsp_50_ (d) and PArg_100_/PGlu_50_ (e) assemblies. The average value is shown as a red diamond. The box plot rectangle indicates the interquartile range between the first and third quartiles, the whiskers delimitate the minimum and maximum distance value (excluding outliers), the horizontal line indicates the median value.

To test whether PAsp or PGlu chains adopt α‐helical structures, we performed MD simulations of alternative hexagonal models starting from PArg_100_ and PAsp_100_ (or PGlu_100_) α‐helical chains. The results showed that, while PArg_100_ chains remained mostly helical, the PAsp_100_ or PGlu_100_ α‐helices quickly transitioned to random coils (Figures S23 and S24). These results are in line with literature data [33], which suggests that polypeptides composed of acidic residues should fold as random coils, as opposed to PArg, which generally adopts an α‐helical structure due to the farther distance between the charged groups of consecutive arginine residues. Additional ESMFold [34] predictions on PArg_100_, PAsp_50/100_, and PGlu_50/100_ sequences confirmed this conclusion. Therefore, these results corroborate the chosen interaction model (PArg_100_ α‐helices in complex with PAsp_50_ or PGlu_50_ extended coils).

The analysis of salt bridges formed between arginine residues and negatively charged residues reveals that while an ideal 1:1 interaction ratio is predominant, a single acidic residue is also often involved in a shared interaction with two, or seldomly, three arginine side chains (Figure S25). A salt bridge lifetime analysis (Figure S26) also shows that most salt bridges are short‐lived (maximum lifetime of 1–10 ns), while a small proportion persists for longer times (100–300 ns).

## 11 Similarities of Structure and Properties to Collagen

Our study reveals many notable similarities between the structure and properties of ordered PArg polypeptide complexes and collagen. Collagen is the predominant structural protein of connective tissue such as cartilage, bone, tendon, ligaments, or skin. It consists of a hierarchical assembly of triple helices forming fibers that provide mechanical strength to tissue [35, 36]. Collagen has widespread applications in tissue regeneration, sustained drug delivery, absorbable surgical sutures, as grafts for tissue engineering, and as the replacement of tendons, bones, blood vessels, and skin [37]. Most of the collagen used for these applications is extracted from animal sources. Its use bears risks due to adverse immunogenic or allergic reactions, potential transmission of diseases or pathogens, and variability of the material depending on the extraction method. Therefore, there have been approaches to synthesize collagen‐mimetic materials. These are synthetic or bio‐engineered systems designed to replicate the structure, self‐assembly, and function of native collagen, particularly its triple‐helical polypeptide structure and the ability to form fibrillar networks [38, 39, 40, 41, 42, 43, 44, 45].

The similarities of PArg complexes and collagen are related to the formation of hierarchical structures based on assemblies of stable long helices. For collagen this is the triple helix, for the polyamino acid complexes this is a polyelectrolyte charge‐stabilized PArg α‐helix. For the PArg complexes, the long, extended helices assemble into long‐range ordered hexagonal or quadratic assemblies, for collagen the triple helices form near‐hexagonal assemblies. Both helix types show periodic ordering along their helical axis. For collagen, this is caused by a staggered position of the polypeptide chains in the triple helix, with a periodicity of 68 nm. For PArg complexes, the long‐period is equal to the fully extended helix length, varying between 15 and 30 nm depending on the PArg molecular weight. In the case of type‐II collagen, the helical assemblies melt via a helix‐coil transition at 41°C [46], the PArg complexes melt at 41°C in the case of PArg_100_‐, and at 53°C in the case of Parg_200_‐ complexes. The helix assemblies can be highly oriented for both systems. For PArg complexes, alignment can be induced by shear, for collagen this is performed by fibroblast tensile forces. Mechanically stable materials can in both cases be obtained by covalent cross‐linking, which for PArg‐complexes was achieved by imide formation using glutardialdehyde. Collagen fibers are crosslinked by enzymatic ester formation. For a crosslinked PArg‐complex we observe a tensile modulus of 6 MPa and a tensile strength of 0.3 MPa, which already close to collagen in articular cartilage with tensile moduli of 5 – 25 MPa and tensile strengths of 0.8 – 25 MPa [47]. Table 2 summarizes the common structures and properties of PArg‐complexes and collagen. Since the PArg polypeptide complexes can be simply fabricated by spontaneous self‐assembly in aqueous solution, and can be easily processed into sheets, films, fibers and coatings, they are a potential alternative to collagen‐based materials.

**TABLE 2:** Similarities between ordered PArg complexes and collagen.

Property	Polyaminoacid Complex	Collagen
Helix‐type	charge‐stabilized α‐helix	triple helix
Helix packing	hexagonal, quadratic	near hexagonal
Long period along axis	equal to extended α‐helix length, 15 nm (100 aa), 30 nm (200 aa)	staggering determined by amino acid sequence, 68 nm
Melting temperature	41°C (100 aa), 53°C (200 aa)	41°C (type II).
Helix‐orientation	via shear	via fibroblast tensile forces
Cross‐linking	imide formation (glutardialdehyde)	ester formation (enzymatic)
Tensile modulus, tensile strength	6 MPa, 0.3 MPa	3 – 8 MPa, 0.2 – 0.4 MPa (articular cartilage)
Water uptake	∼ 20 wt.%	50 – 70 wt.%

## 12 Conclusions

We demonstrate for polyarginine polypeptides that the addition of oppositely charged polyamino acids such as polyaspartic acid and polyglutamic acid at low and high salt concentrations leads to a liquid‐solid phase separation with the spontaneous formation of highly ordered solid complexes, a rare case of the formation of ordered polyelectrolyte complexes. The complexes exhibit a hierarchical structure based on extended PArg α‐helices that are charge‐stabilized by PAsp or PGlu‐chains. The extended PArg α‐helices form long‐range ordered hexagonal or square packed arrays with an additional periodic staggering along their helical axis. The corresponding repeat distance is in the range of 15 – 30 nm, equal to the length of the extended α‐helices. The helices can be shear‐aligned to achieve macroscopic uni‐axial helical orientation. Crosslinking leads to stable, solid elastic materials. Upon heating to temperatures above 40°C the complexes melt and disorder due to a reversible helix‐coil transition. The polyarginine complexes therefore assemble into a highly ordered, hierarchical helix/ structure very similar to collagen and show very similar properties. Since the complex structure can be stabilized by covalent cross‐linking, they are potentially very suitable as synthetic collagen‐mimetic materials.

## Author Contributions

**Benoit David**: investigation, Writing – original draft, Writing – review and editing, formal analysis. **Karoline Rengel**: investigation, formal analysis. **Beate Förster**: investigation, formal analysis. **Henrike Heise**: investigation, methodology, formal analysis. **Ralf Biehl**: methodology, formal analysis. **Michael Ohl**: investigation, formal analysis. **Ekaterina Buvalaia**: investigation, formal analysis, writing – original draft. **Stephan Förster**: conceptualization, writing – original draft, supervision. **Margarita Kruteva**: methodology, formal analysis. **Martin Dulle**: investigation, formal analysis. **Holger Gohlke**: supervision, conceptualization.

## Conflicts of Interest

The authors declare no conflicts of interest.

## Supporting information




**Supporting File**: smll75495‐sup‐0001‐SuppMat.pdf.

## Data Availability

The data that support the findings of this study are available on request from the corresponding author. The data are not publicly available due to privacy or ethical restrictions.
