# Supplementary material for: Oppositely Charged Homopolypeptides Form Ordered Helix Assemblies With Hierarchical Structure
Source: Small. 2026 Sep 11;22(62):e75495. doi: 10.1002/smll.75495 (PMC13742891; doi:10.1002/smll.75495)
Supplement: Supplementary file 1 [file SMLL-22-e75495-s001.pdf]

Supplementary Information

Oppositely charged homopolypeptides form ordered helix assemblies with a hierarchical collagen-mimetic structure

Ekaterina Buvalaia[1], Martin Dulle[1], Benoit David[2], Karoline Rengel[1], Michael Ohl[1], Beate Förster[3], Margarita Kruteva[1], Henrike Heise[4,5], Ralf Biehl[1], Holger Gohlke[2,6], Stephan Förster[1,7*]

[1] JCNS-1, Forschungszentrum Jülich, 52428 Jülich, Germany
[2] IBG-4, Forschungszentrum Jülich, 52428 Jülich, Germany
[3] ERC-1, Forschungszentrum Jülich, 52428 Jülich, Germany
[4] Institute for Physical Chemistry, Heinrich Heine University Düsseldorf, 40225 Düsseldorf, Germany
[5] IBI-7, Forschungszentrum Jülich, 52428 Jülich, Germany
[6] Institute for Pharmaceutical and Medicinal Chemistry, Heinrich Heine University Düsseldorf, 40225 Düsseldorf, Germany
[7] Institute of Physical Chemistry, RWTH Aachen University, 52074 Aachen, Germany

1. Samples …………………………………………………………………………….2
2. Optical microscopy of solid and liquid complexes ………………………………...…3
3. SAXS-investigations of solid complexes ……………………………………………4
4. Shear-alignment ……………………………………………………………………..13
5. Helical assembly model …………………………….……………………………14
6. Results of MD simulations …………………………………………………………15
7. NMR investigations of phase separated complexes ……………………………...25
8. ^{13}C ssNMR of polyaminoacid conformation ………………………………………..26

1. Samples

Table S1. Polyaminoacids investigated in the present study. For the polyarginines (PArg) the molecular weights are given for the respective hydrochlorides. For polystyrene sulfonic acid (PSSH), we give the number of repeat units. The molecular weights were determined by NMR by the supplier (Alamanda Polymers). The polydispersities were determined by SEC to be in the range of 1.00 – 1.20 for all samples, according to the supplier.

	Average Molecular Weight [g/mol]	Average number of repeat units [aa]	Molecular Weight Range [g/mol]	Repeat unit Range [aa]
$PArg_{30}$	5800	30	5600 – 6400	27 – 33
$PArg_{50}$	9600	50	8700 – 10600	45 – 55
$PArg_{100}$	19000	100	17300 – 21200	90 – 110
$PArg_{200}$	38500	200	34600 - 42400	180 - 220
$PAsp_{100}$	11500	100	10300 – 12700	90 - 110
$PGlu_{100}$	13000	100	11600 – 14200	90 – 110
PSSH	17500	95		

2. Optical microscopy of solid and liquid complexes

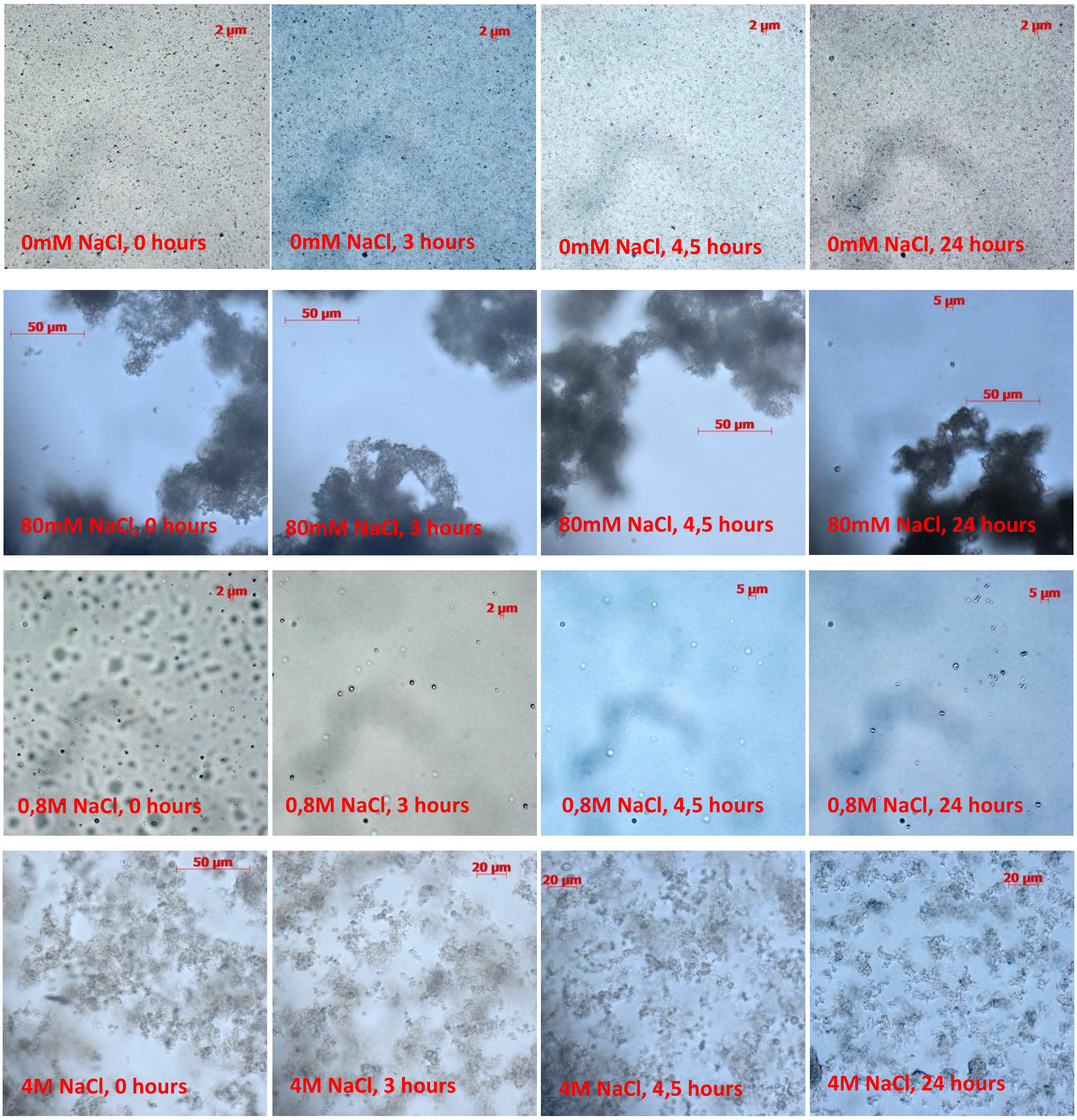

Figure S1. Optical microscopy images of PArg/PAsp complexes formed at different NaCl concentrations directly after preparation and after 3, 4, 5 and 24 hours, at 0.7mM (10,7 mg/ml) total polymer concentration.

3. SAXS investigations of solid complexes

For all SAXS curves the empty cell scattering has been subtracted. A salt concentration of 0 mM refers to complexes that were prepared without added salt.

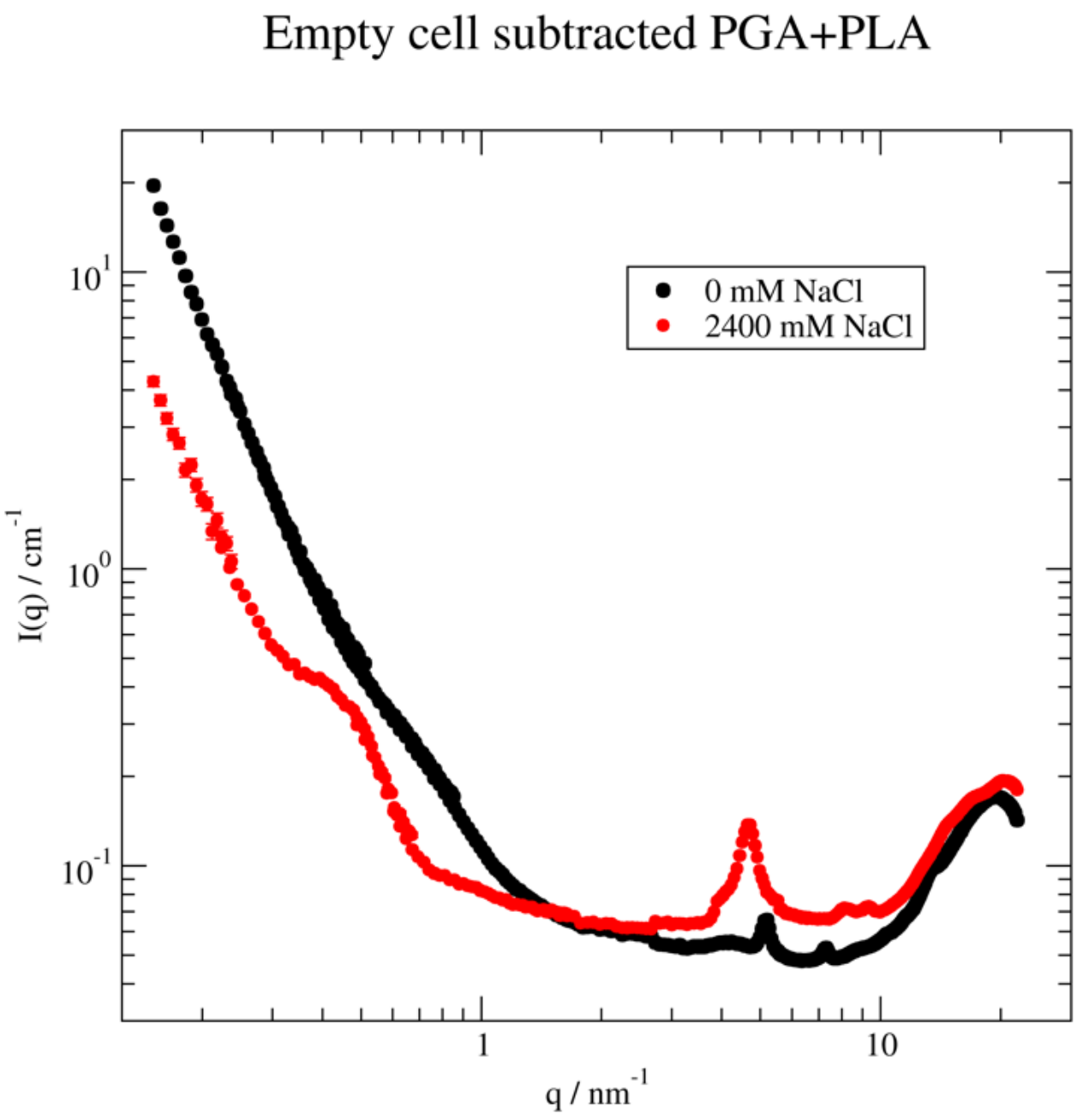

Figure S2. Measured SAXS curves for $PArg_{100}/PGlu_{100}$ complexes prepared at NaCl concentrations 0 mM (black) and 2400 mM (red).

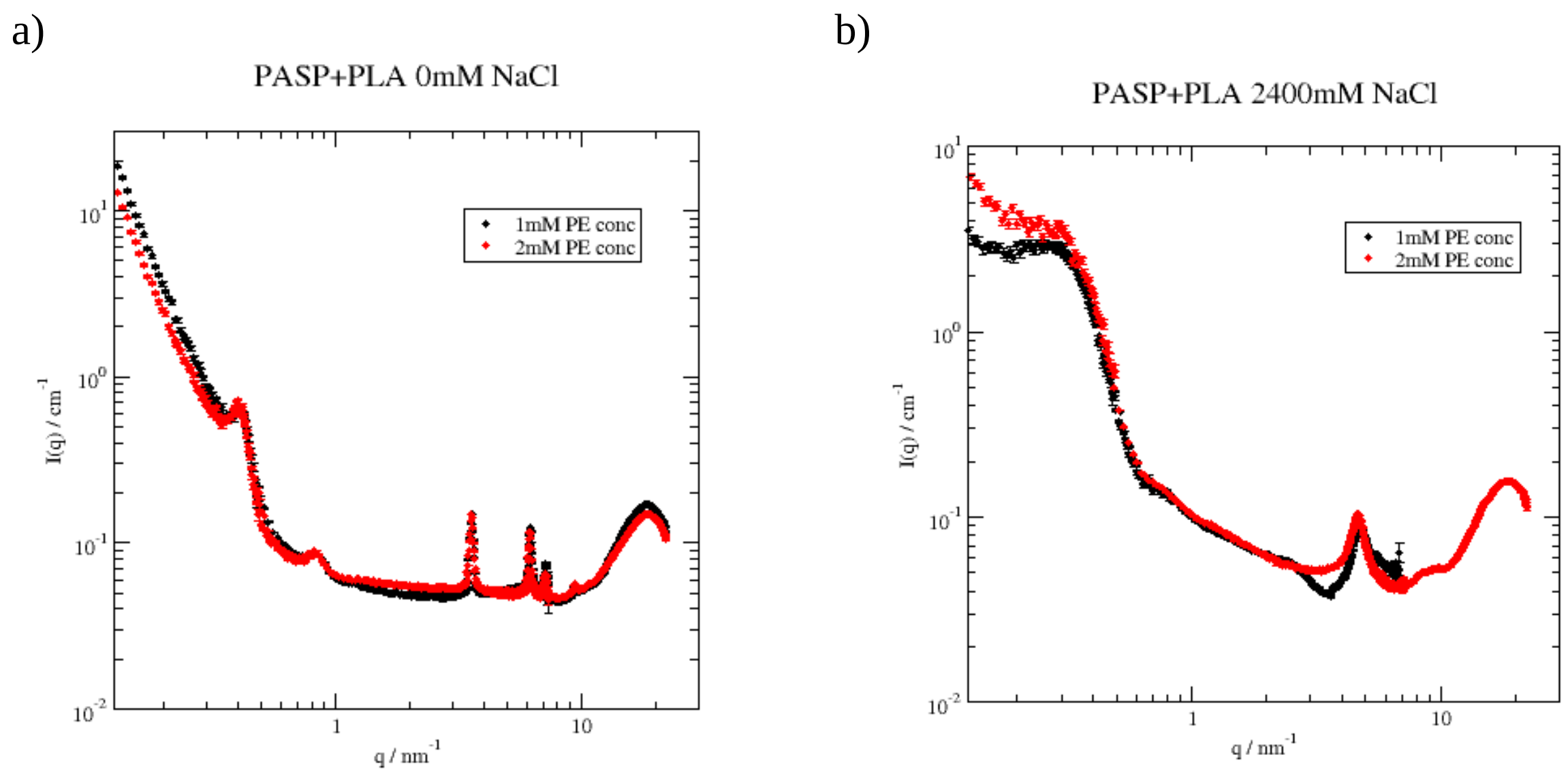

Figure S3. Comparison of measured SAXS curves for $PArg_{100}/PAsp_{100}$ complexes prepared at 1 mM (black) and 2 mM (red) polymer concentration at a) NaCl concentrations of 0 mM and b) NaCl concentrations of 2400 mM.

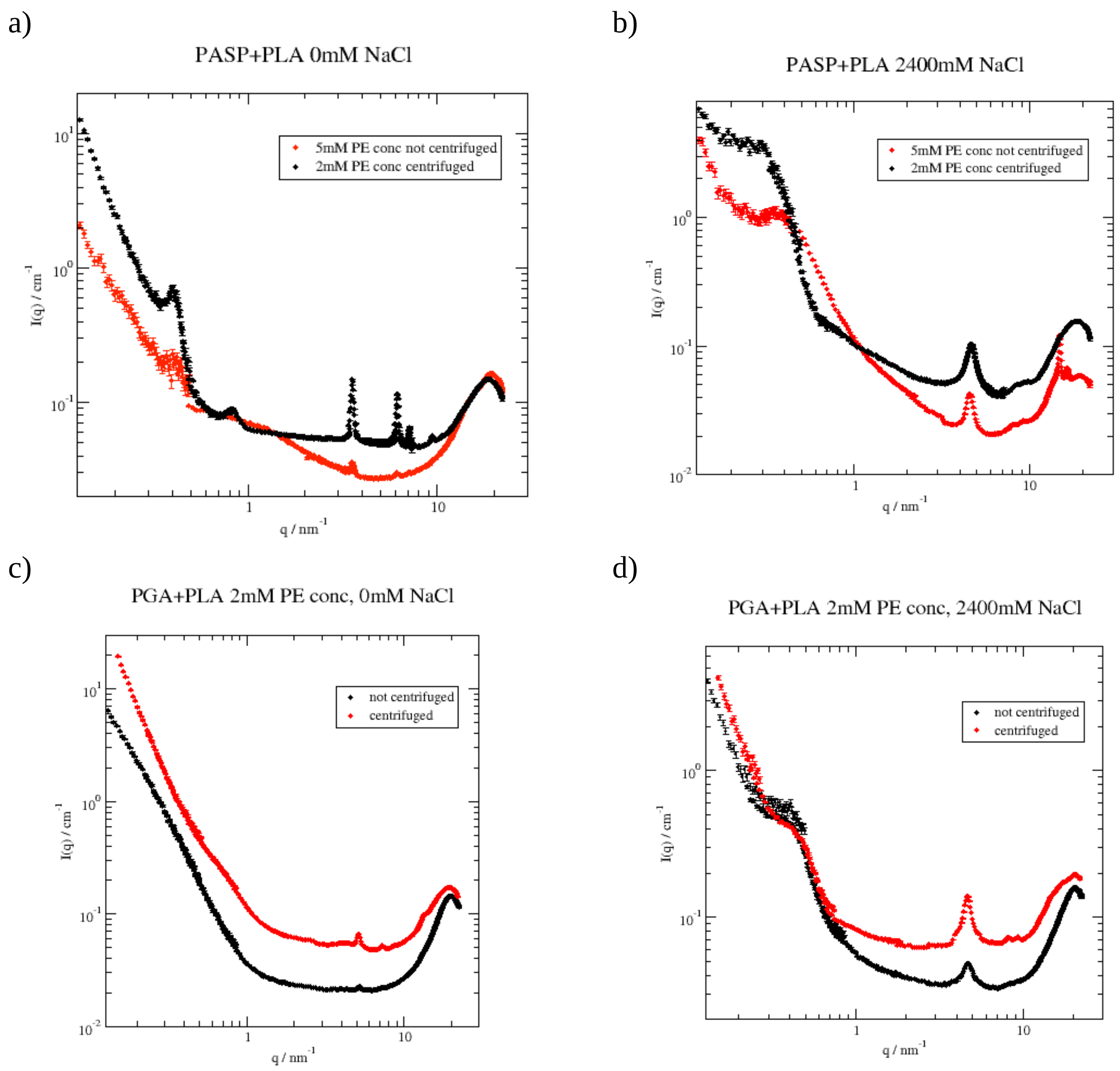

Figure S4. Comparison of measured SAXS curves for $PArg_{100}/PAsp_{100}$ complexes (a, b) and $PArg_{100}/PGlu_{100}$ complexes (c, d) prepared without centrifugation (black) and with centrifugation (red) at salt concentrations of 0 mM NaCl (a, c) and 2400 mM NaCl (b, d).

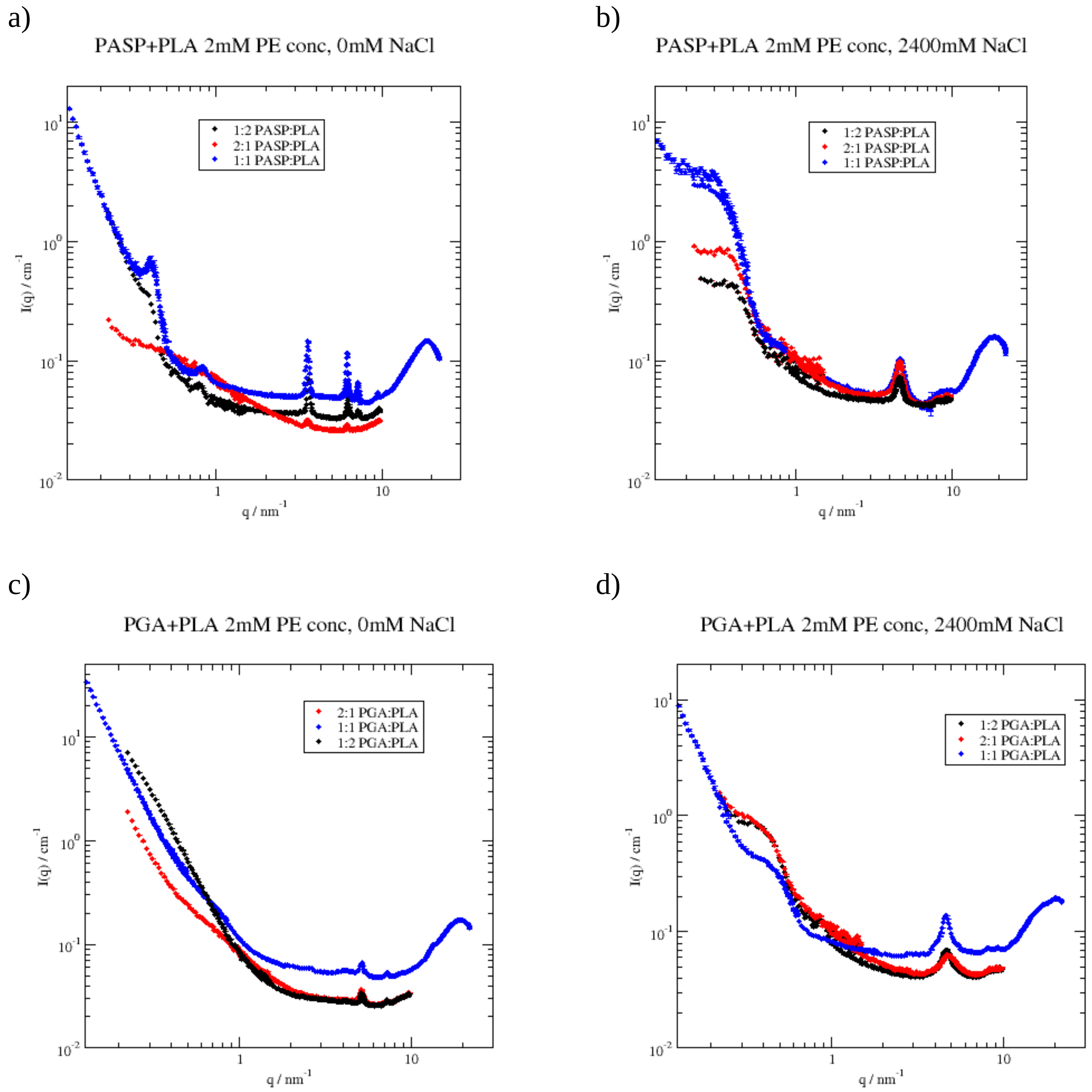

Figure S5. Comparison of measured SAXS curves for $PArg_{100}/PAsp_{100}$ complexes (a, b) and $PArg_{100}/PGlu_{100}$ complexes (c, d) prepared at PAsp:PArg- and PGlu:PArg-ratios of 2:1 (red), 1:1 (blue) and 1:2 (black) and salt concentrations of 0 mM NaCl (a, c) and 2400 mM NaCl (b, d).

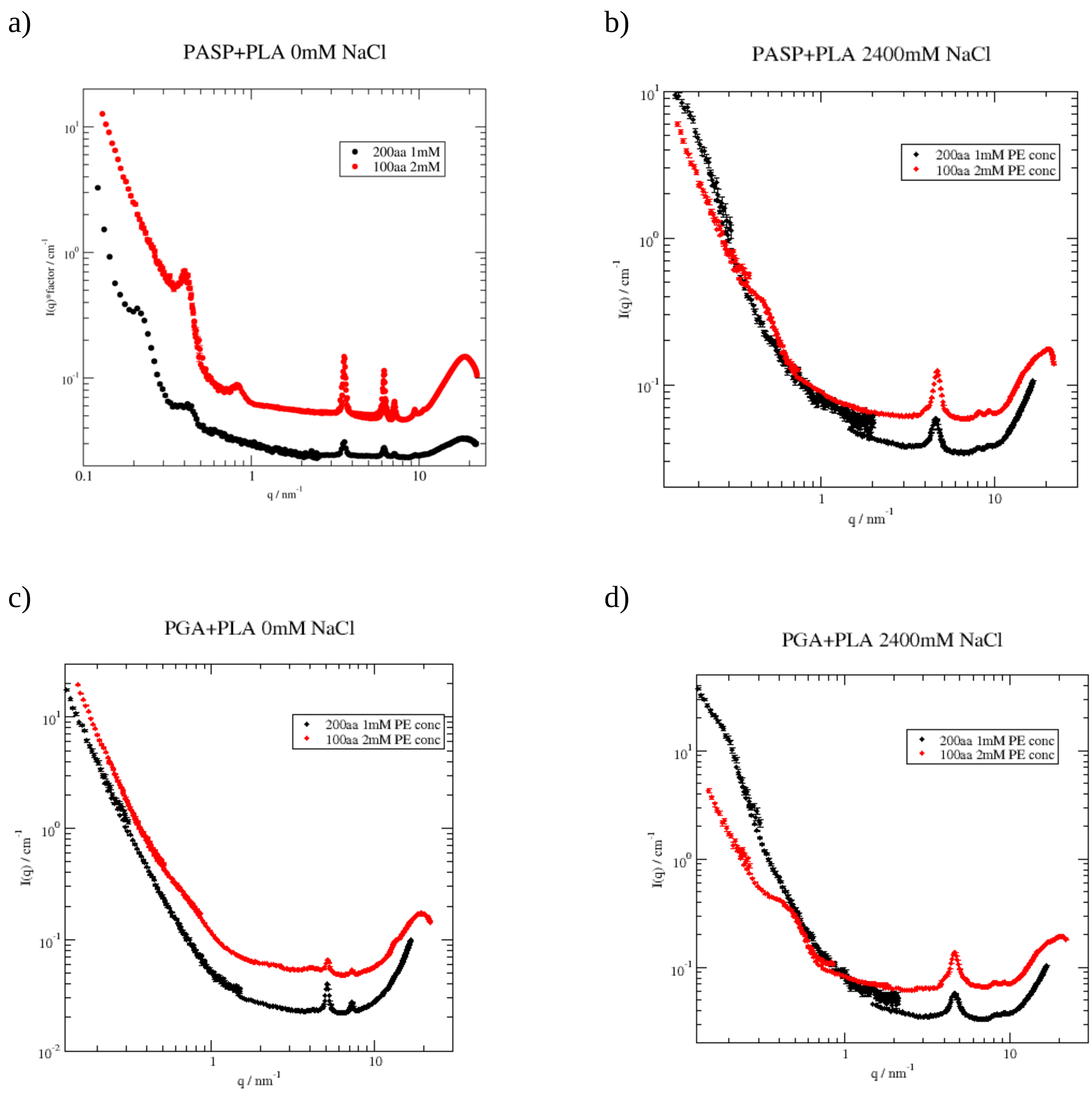

Figure S6. Comparison of measured SAXS curves for $PArg_{100}/PAsp_{100}$ complexes (a, b) and $PArg_{100}/PGlu_{100}$ complexes (c, d) prepared at PArg-chain lengths of 100 aa (red) and 200 aa(black) at salt concentrations of 0 mM NaCl (a, c) and 2400 mM NaCl (b, d).

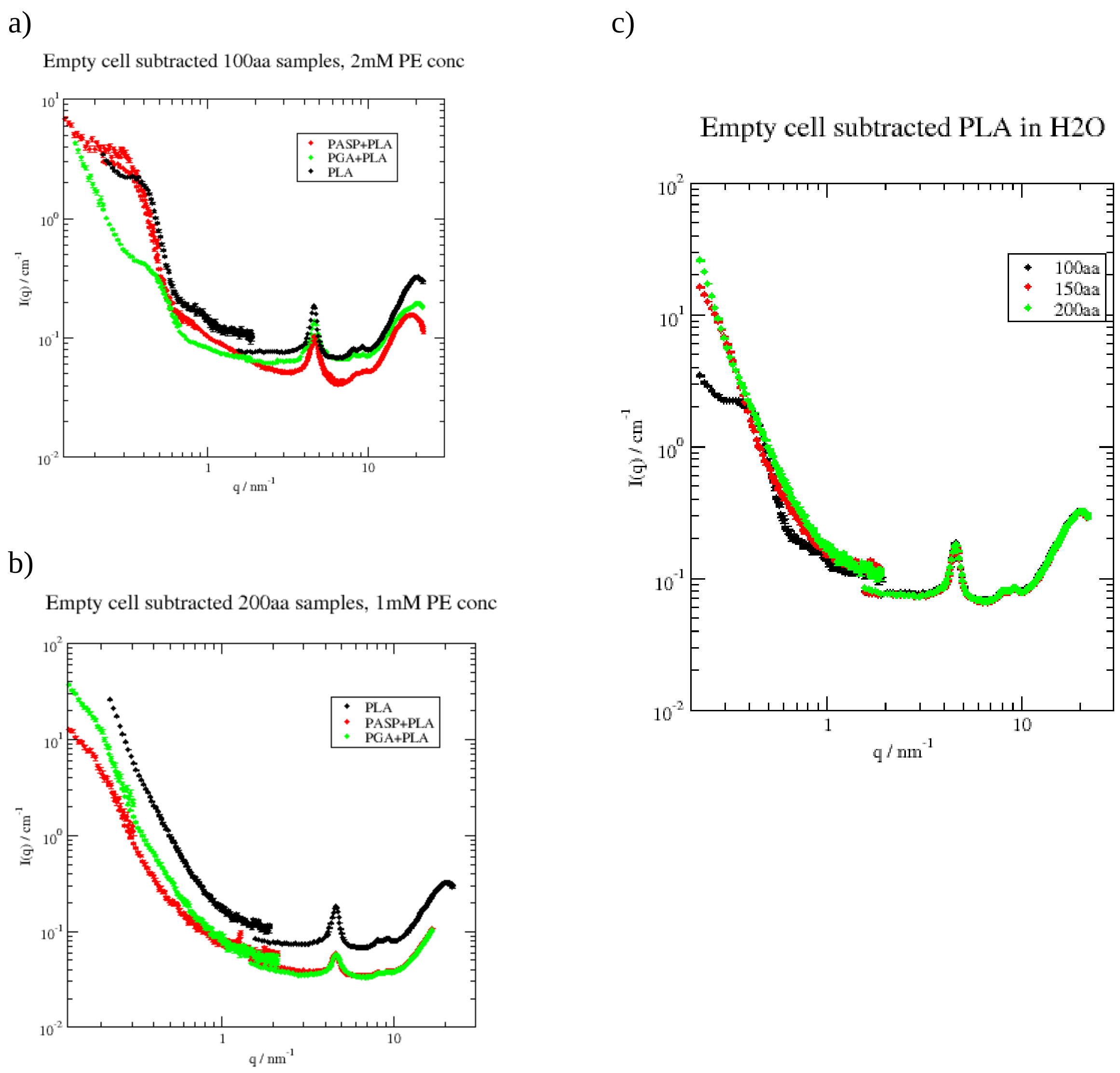

Figure S7. (a/b) Comparison of measured SAXS curves for PArg/PAsp–complexes (red), PArg/PGlu-complexes (green) and PArg (black) prepared at a salt concentration of 2400 mM NaCl for $PArg_{100}$ (a) and $PArg_{200}$ (b). (c) shows the measured SAXS curves for PArg at a salt concentration of 2400 mM NaCl for chain lengths of 100aa (black), 150aa (red), and 200aa (green). We observe that the SAXS curves at high salt concentrations of 2400 mM are the same, irrespective of the composition of the complexes.

a)

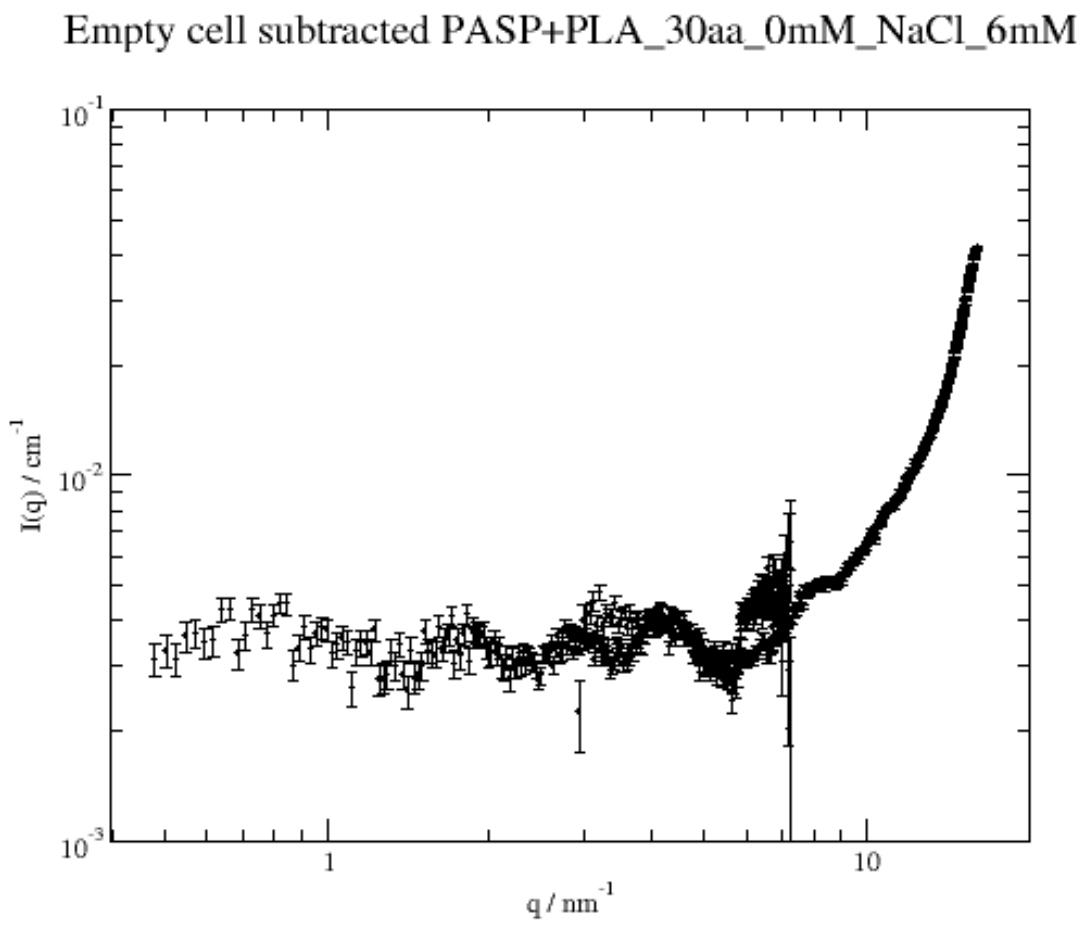

b)

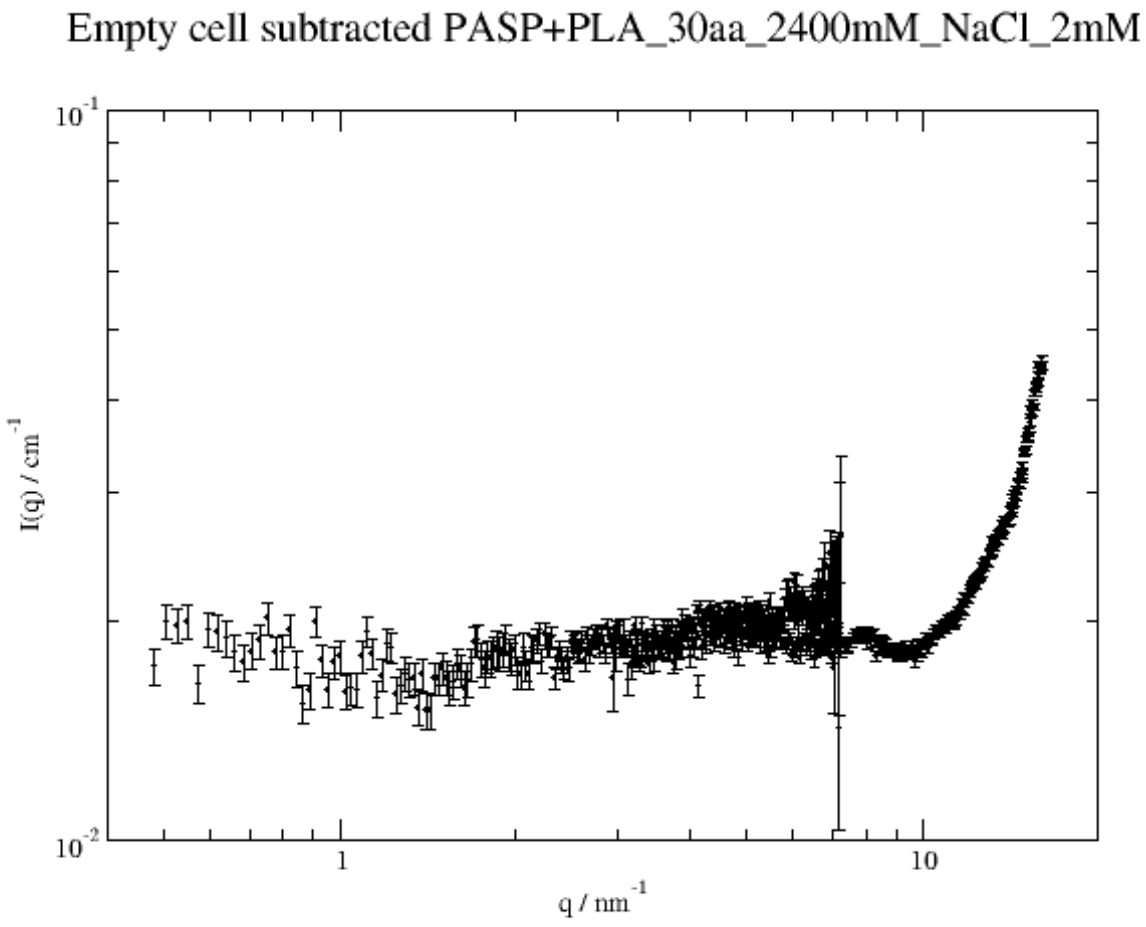

c)

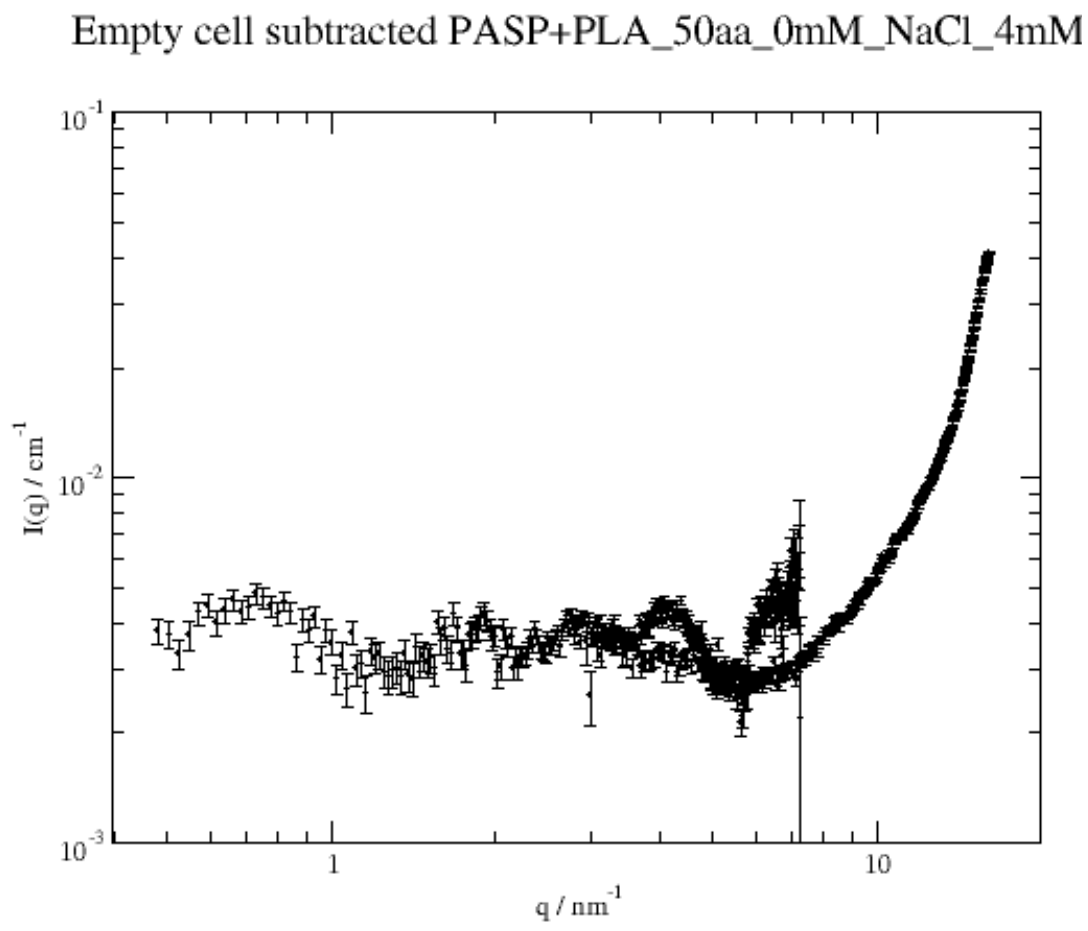

d)

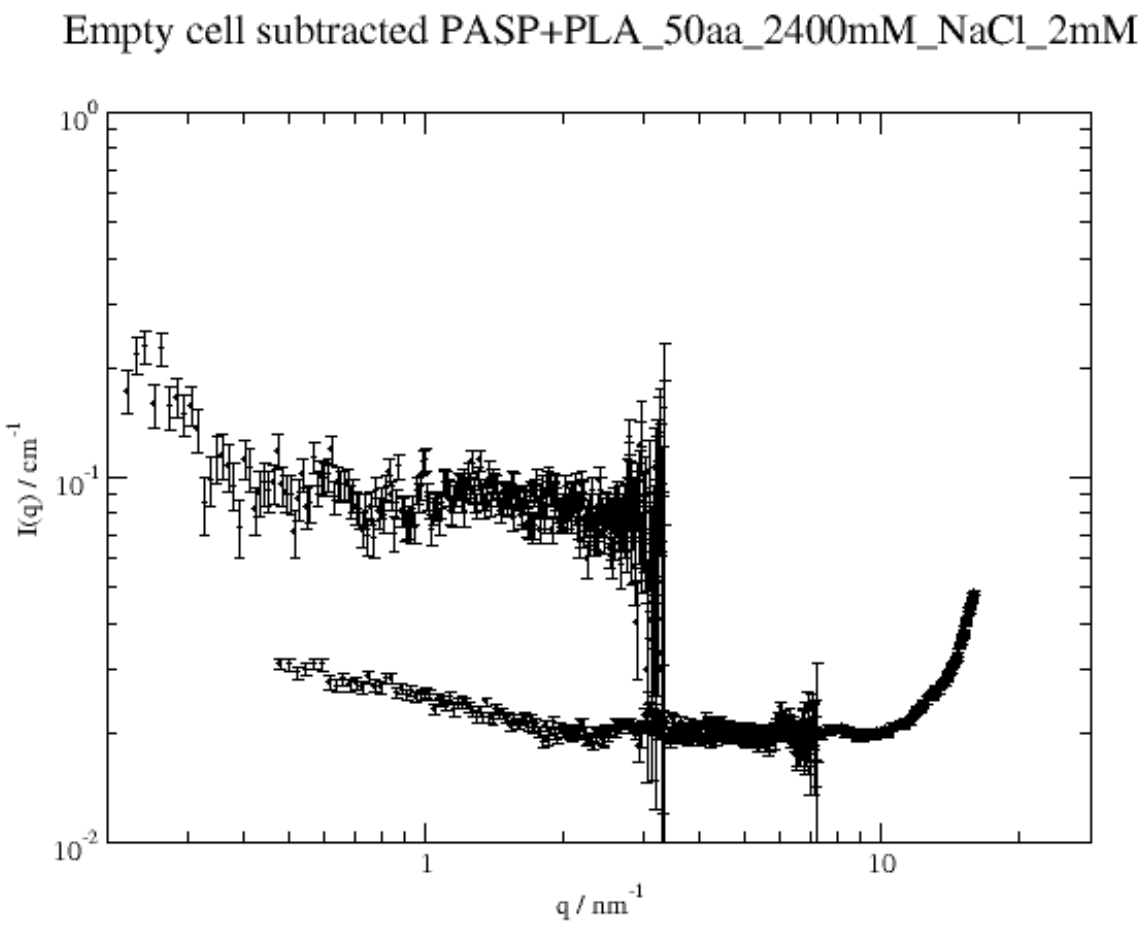

Figure S8. Comparison of measured SAXS curves for PArg/PAsp complexes prepared at PArg-chain lengths of 30 aa (a, b) and 50 aa (c, d) at salt concentrations of 0 mM NaCl (a, c) and 2400 mM NaCl (b, d). For 30 and 50 aa we do not observe the formation of ordered helix structures, irrespective of the salt concentration.

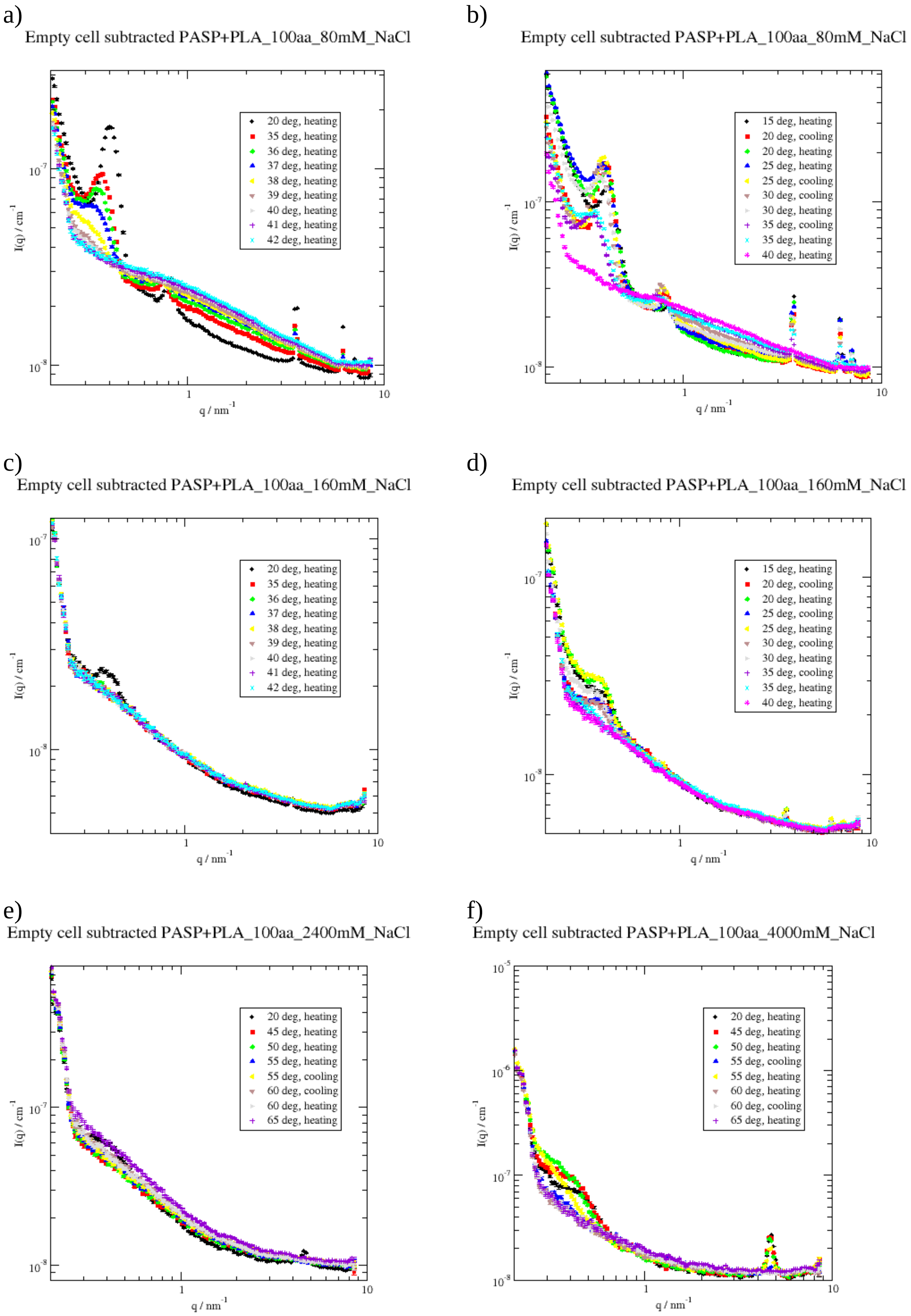

Figure S9. Measured SAXS curves for $PArg_{100}/PAsp_{100}$ during heating to determine the melting temperature (a, c) and during a heating/cooling cycle (b, d, e, f) to demonstrate reversibility of the melting transition. The complexes were prepared at salt concentrations of 80 mM NaCl (a, b), 160 mM NaCl (c, d), 2400 mM NaCl (e) and 4000 mM NaCl [(f).

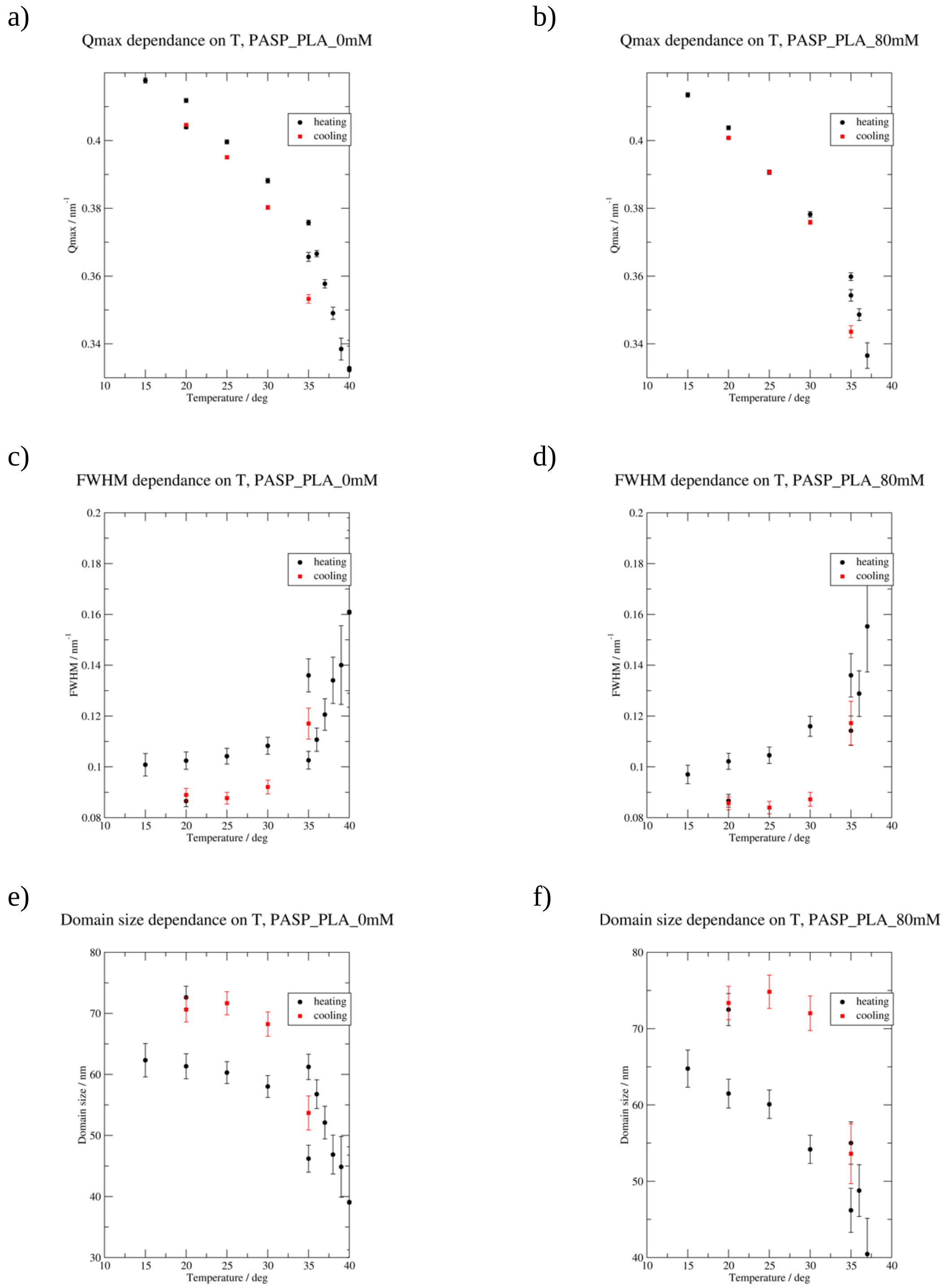

Figure S10. SAXS intermediate primary peak positions q_{max} (a, b), peak width FWHM-values of the primary peaks (c, d) and domain size D = 2π/FWHM (e, f) for $PArg_{100}/PAsp_{100}$ during heating (black) and cooling (red) at salt concentrations of 0 mM NaCl (a, c, e) and 80 mM NaCl (b, d, f).

For the measured temperature-dependent SAXS-curves shown in Fig. S9 the primary peak positions q_{max}, the peak width FWHM-values and the domain sizes D = 2π/FWHM are presented in Fig. S10. For 0 and 80mM NaCl we see a linear decrease of q_{max} with increasing

temperature. FWHM and domain size dependences on temperature change their behavior at the melting temperature. During cooling the domain size is bigger than during heating, indicating that larger crystals grow after this process.

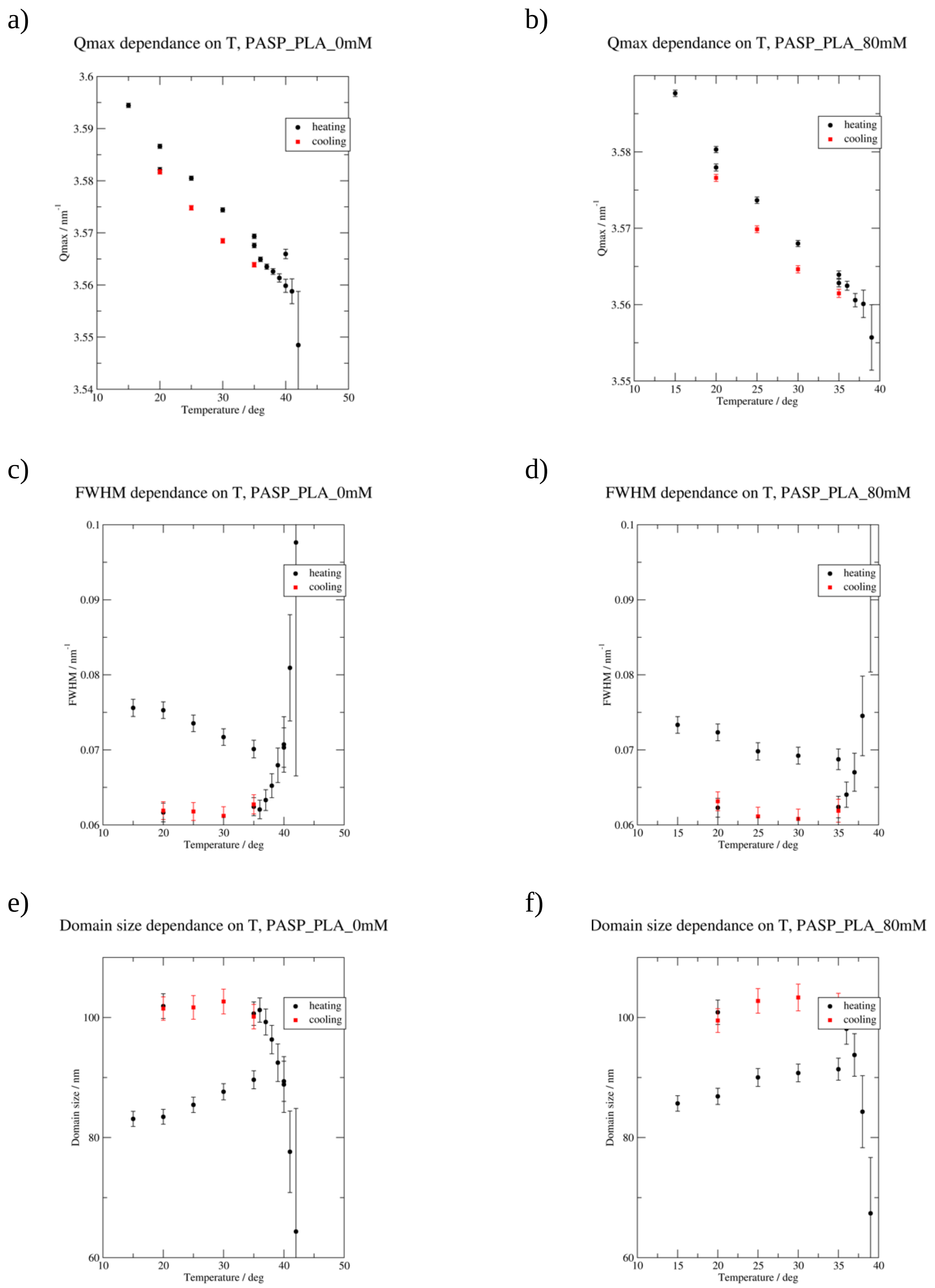

Figure S11. SAXS low-q primary peak positions q_{max} (a, b), peak width FWHM-values of the primary peaks (c, d) and domain size $D = 2\pi/FWHM$ (e, f) for $PArg_{100}/PAsp_{100}$ during heating (black) and cooling (red) at salt concentrations of 0 mM NaCl (a, c, e) and 80 mM NaCl (b, d, f).

4. Shear alignment

The shear alignment was done in a home-built co-axial capillary device with x mm outer diameter and y mm inner diameter.

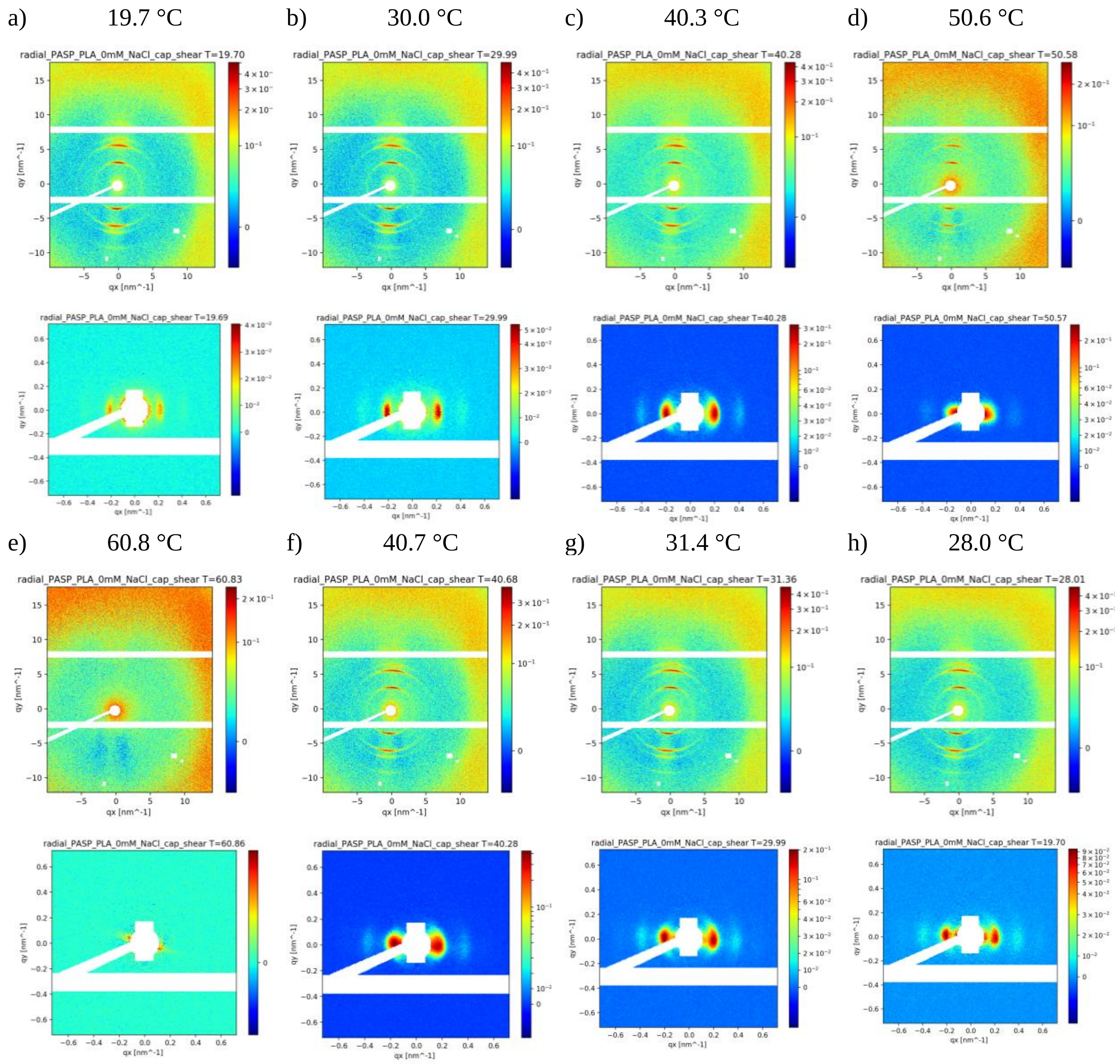

Figure S12. SAXS patterns in the mid-*q*-region (upper panels) and the low-*q* region (lower panels) measured for shear-oriented $PArg_{200}/PAsp_{100}$-complexes (0 mM NaCl) as a function of increasing (a – e) and decreasing temperature (f – h). Above the melting temperature (e) the interhelix peaks on the meridian and the long-range peaks on the equator both disappear. They surprisingly reappear upon cooling.

5. Helical assembly model

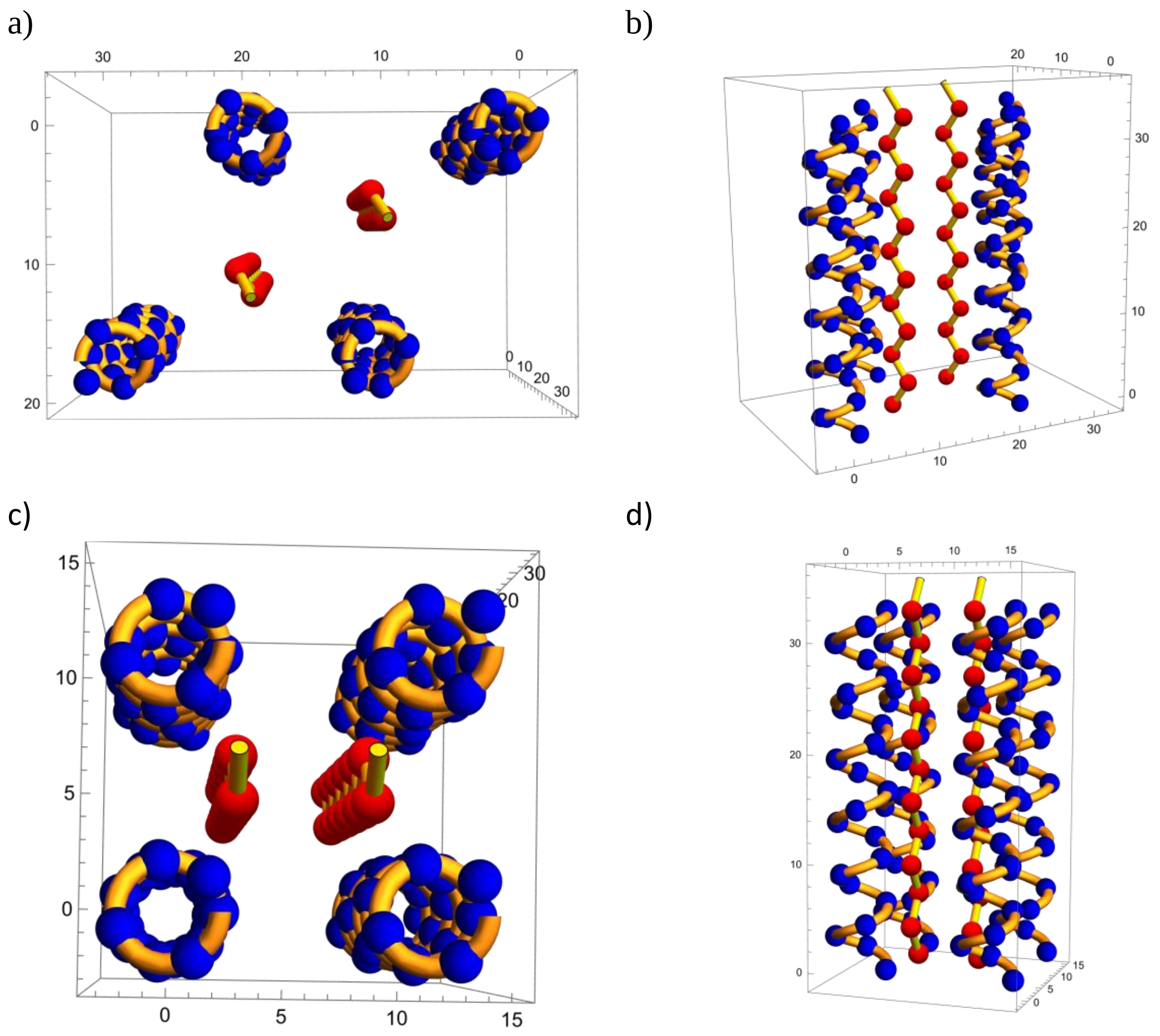

Figure S13. Idealized structural schemes of the hexagonal (a,b) and square (c,d) unit cells with the backbones of the PArg-helices and PAsp- or PGlu-chains indicated.

6. Results of MD simulations

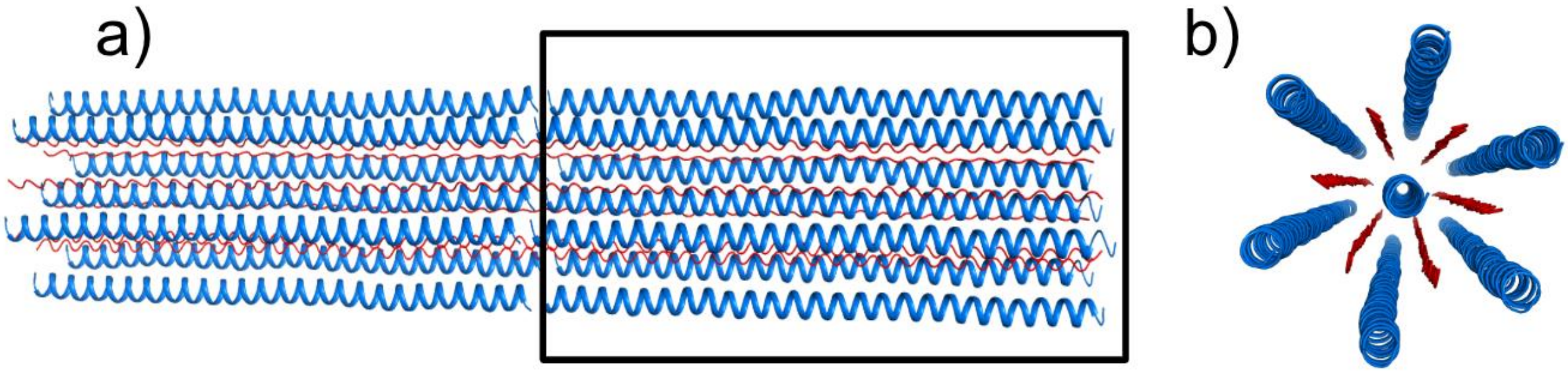

Figure S14. Hexagonal model involving acidic polyaminoacid chains modeled as extended coils. a) Putative interaction model for $PArg_{100}/PAsp_{100}$ (or $PArg_{100}/PGlu_{100}$): head-to-tail interaction of pairs of $PArg_{100}$ α-helices with $PAsp_{100}$ (or $PGlu_{100}$) extended coils. Black frame: minimal model used for MD simulations, i.e. $PArg_{100}$ α-helices in complex with $PAsp_{50}$ (or $PGlu_{50}$) extended coils. b) View of the hexagonal model along the helix axis.

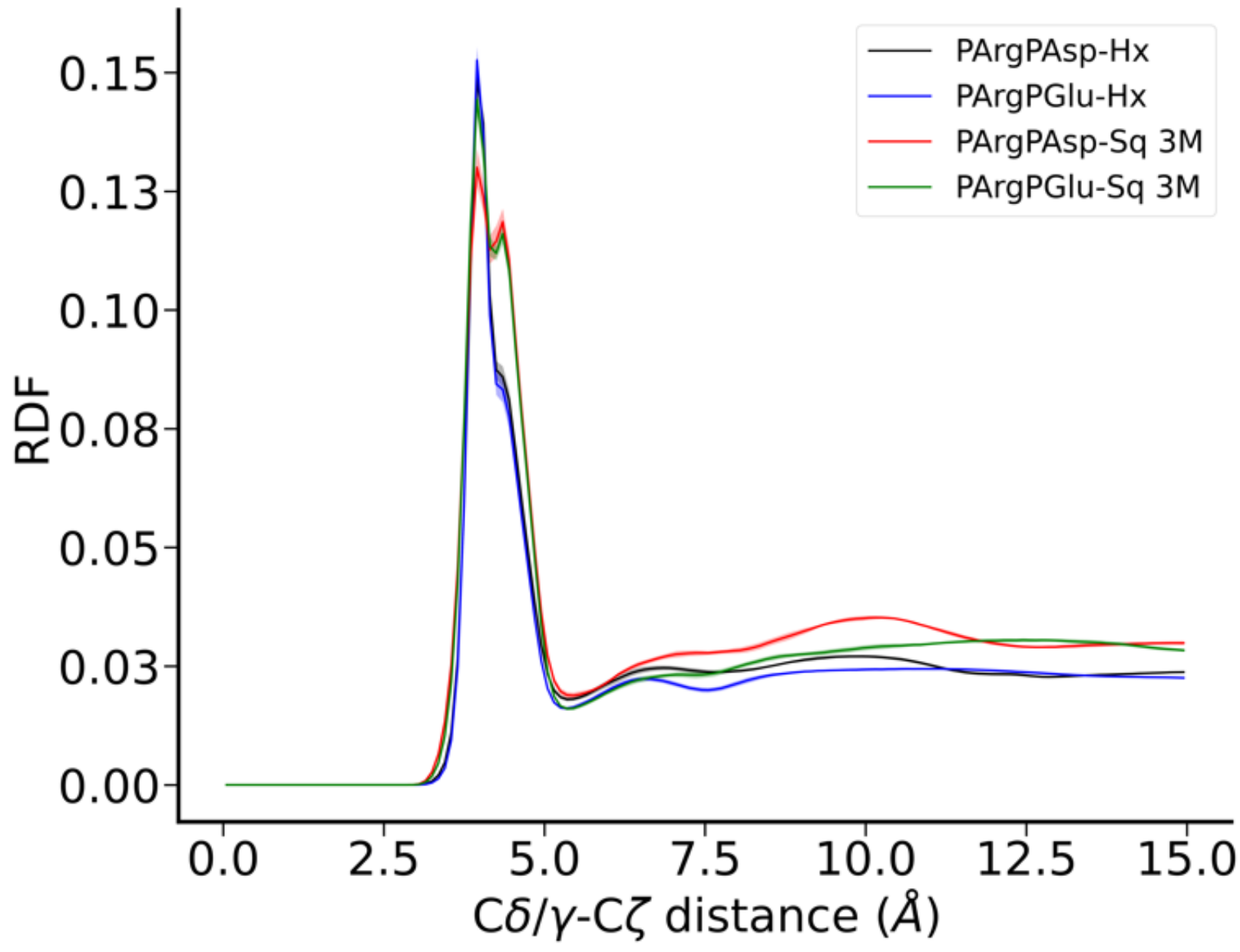

Figure S15 Radial distribution function of distances between carbon atoms of a guanidinium moiety (PArg) and a carboxylate moiety (PAsp/PGlu) in MD simulations. Hx: hexagonal assemblies. Sq 3M: square assemblies simulated in 3 M NaCl.

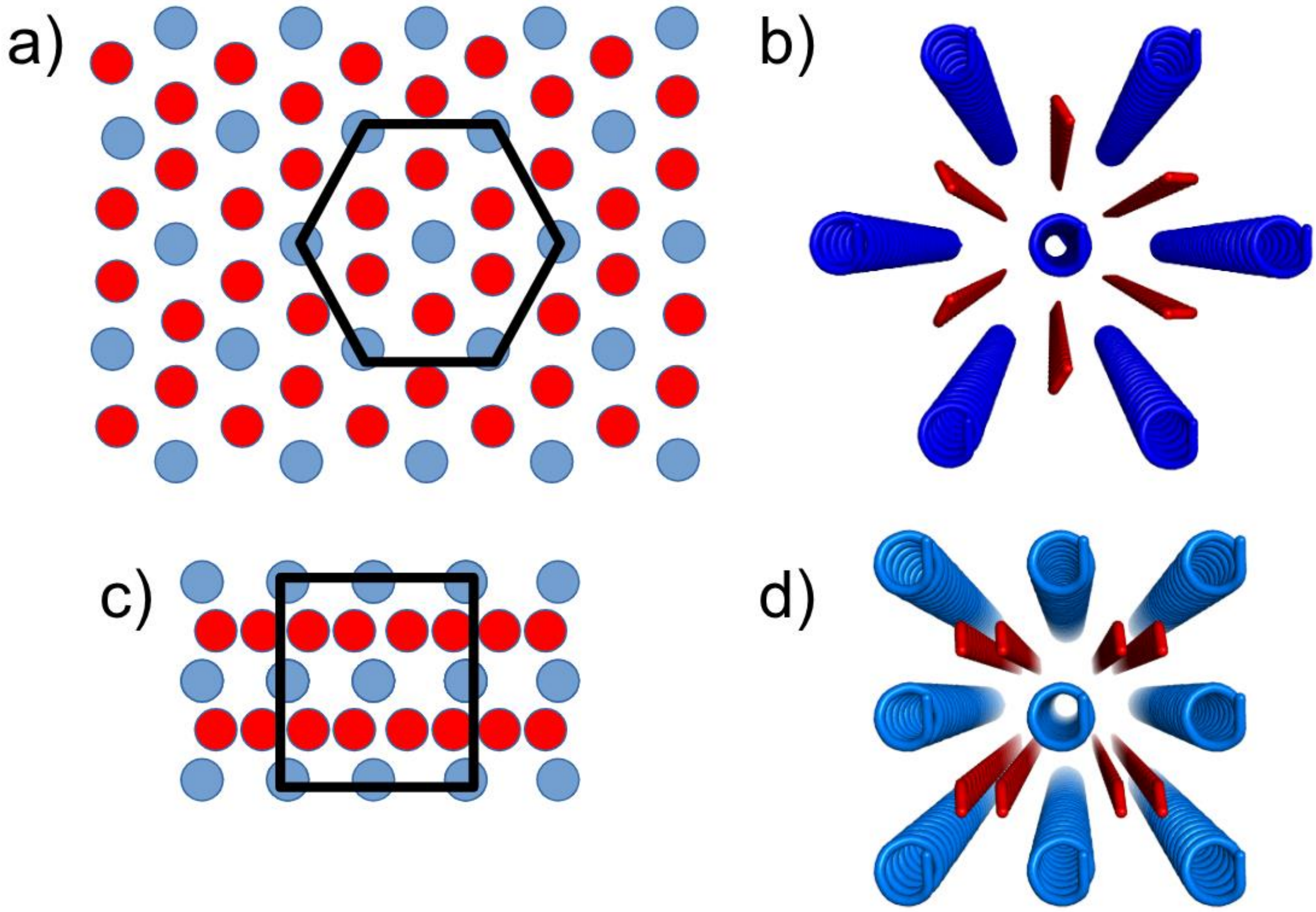

Figure S16 Structural models for the hexagonal and square assemblies used for MD simulations. a) Extended crystal lattice embedding the modeled hexagonal assembly (delimited by a black frame). b) 3D model of the hexagonal assembly built with MODELLER. a) Extended crystal lattice embedding the square assembly (delimited by a black frame). b) 3D model of the square assembly built with MODELLER. Blue: PArg chains. Red: PAsp/PGlu chains.

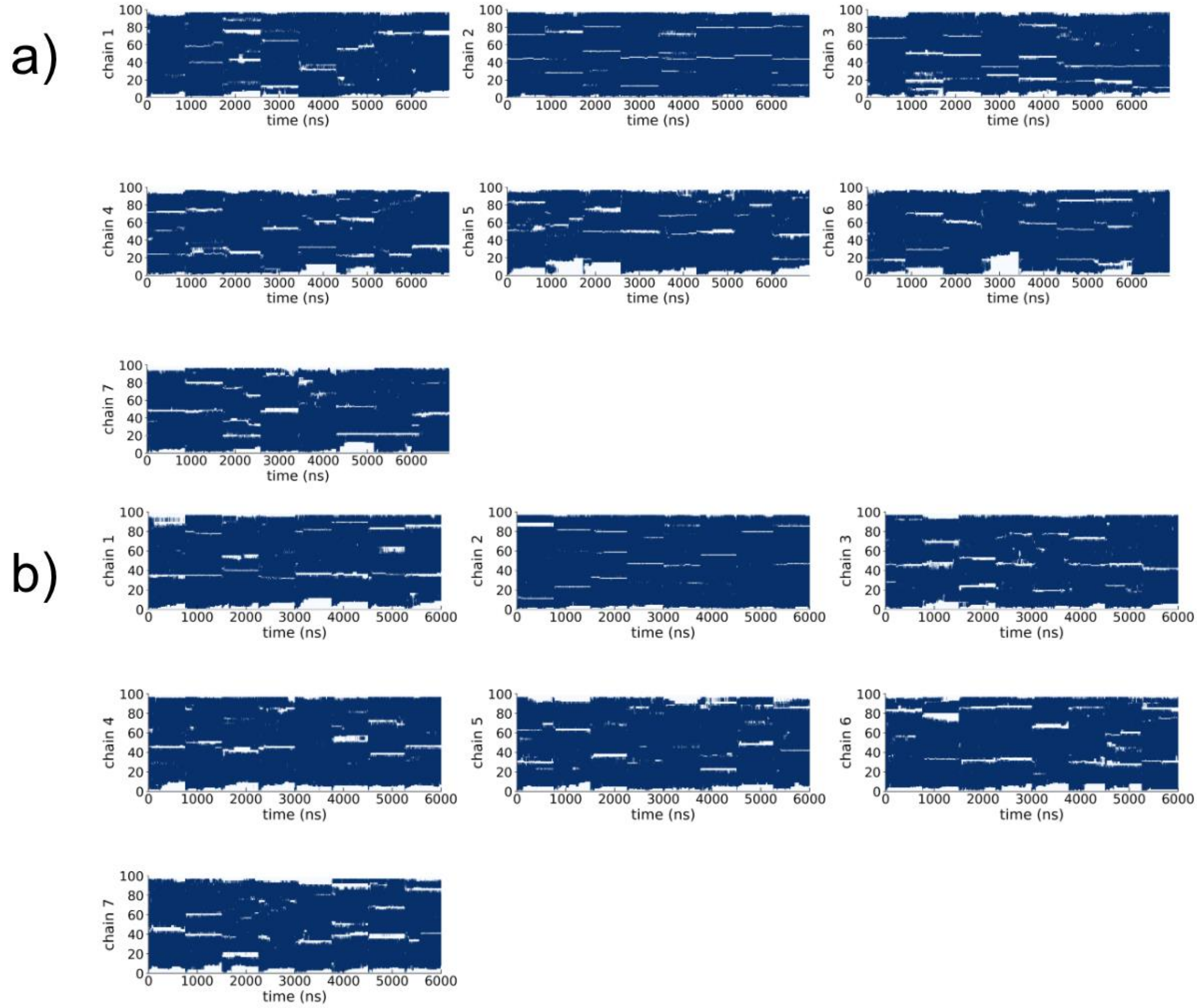

Figure S17 α-helical content of individual $PArg_{100}$ chains predicted by DSSP for a) $PArg_{100}/PAsp_{50}$ and b) $PArg_{100}/PGlu_{50}$ hexagonal assemblies. Blue region: α-helical. White region: random coil. Each plot was generated by concatenating the results obtained for 8 simulation replicas end-to-end.

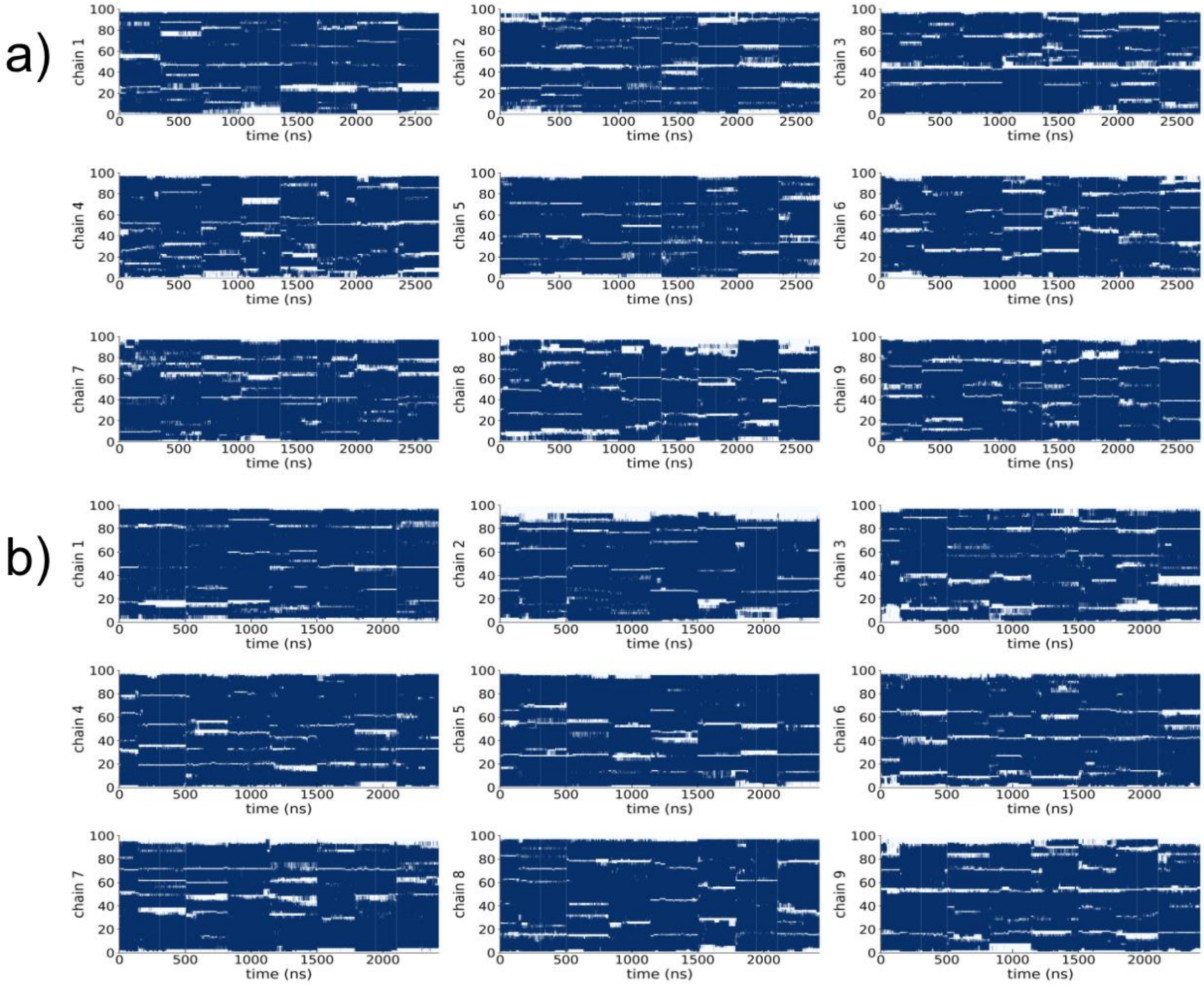

Figure S18 α-helical content of individual $PArg_{100}$ chains predicted by DSSP for a) $PArg_{100}$/$PAsp_{50}$ and b) $PArg_{100}$/$PGlu_{50}$ square assemblies. Blue region: α-helical. White region: random coil. Each plot was generated by concatenating the results obtained for 8 simulation replicas end-to-end.

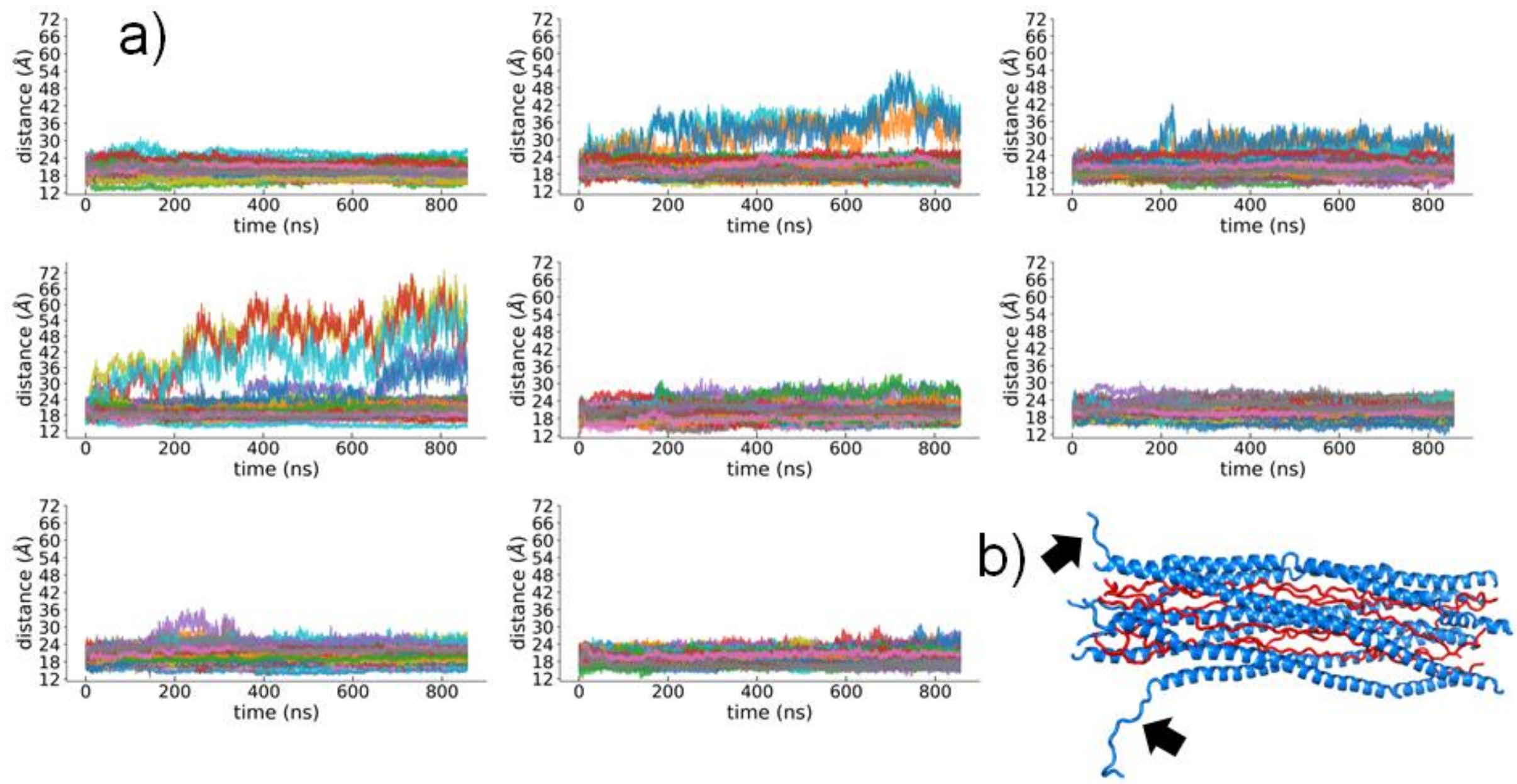

Figure S19 Structural variability of $PArg_{100}$ helices in the $PArg_{100}/PAsp_{50}$ hexagonal model during MD simulations. a) **Cα-Cα** distances (sampled for every tenth residue) between equivalent residues in adjacent $PArg_{100}$ chains. Each plotted line refers to a given **Cα-Cα** distances sampled in one MD simulation replica. b) Extracted snapshot from MD simulations showing local structural unfolding. Black arrows: unfolding of N-termini.

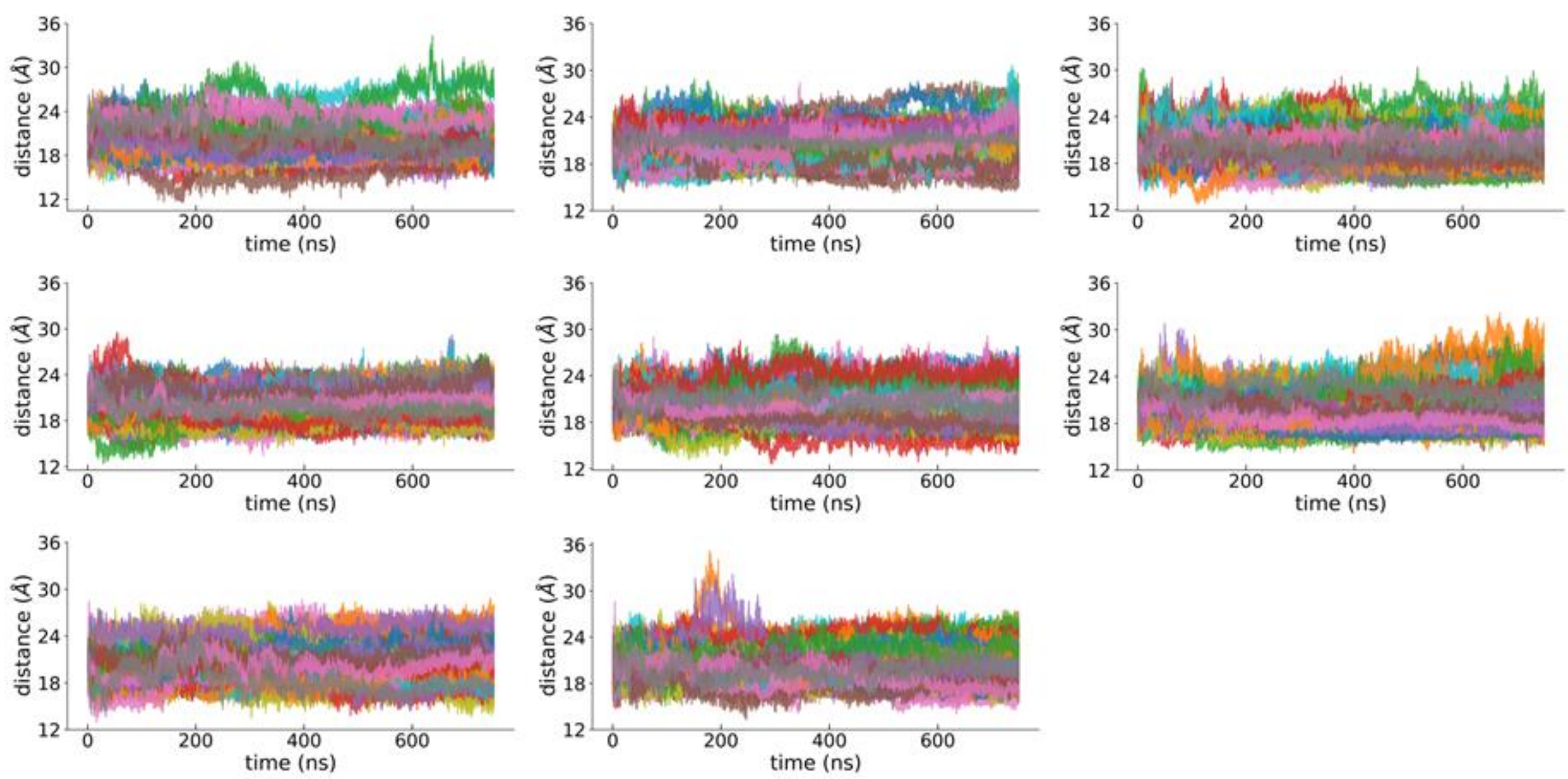

Figure S20 Structural variability of $PArg_{100}$ helices in the $PArg_{100}/PGlu_{50}$ hexagonal model during MD simulations. a) **Cα-Cα** distances (sampled for every tenth residue) between equivalent residues in adjacent $PArg_{100}$ chains. Each plotted line refers to a given **Cα-Cα** distance sampled in one MD simulation replica.

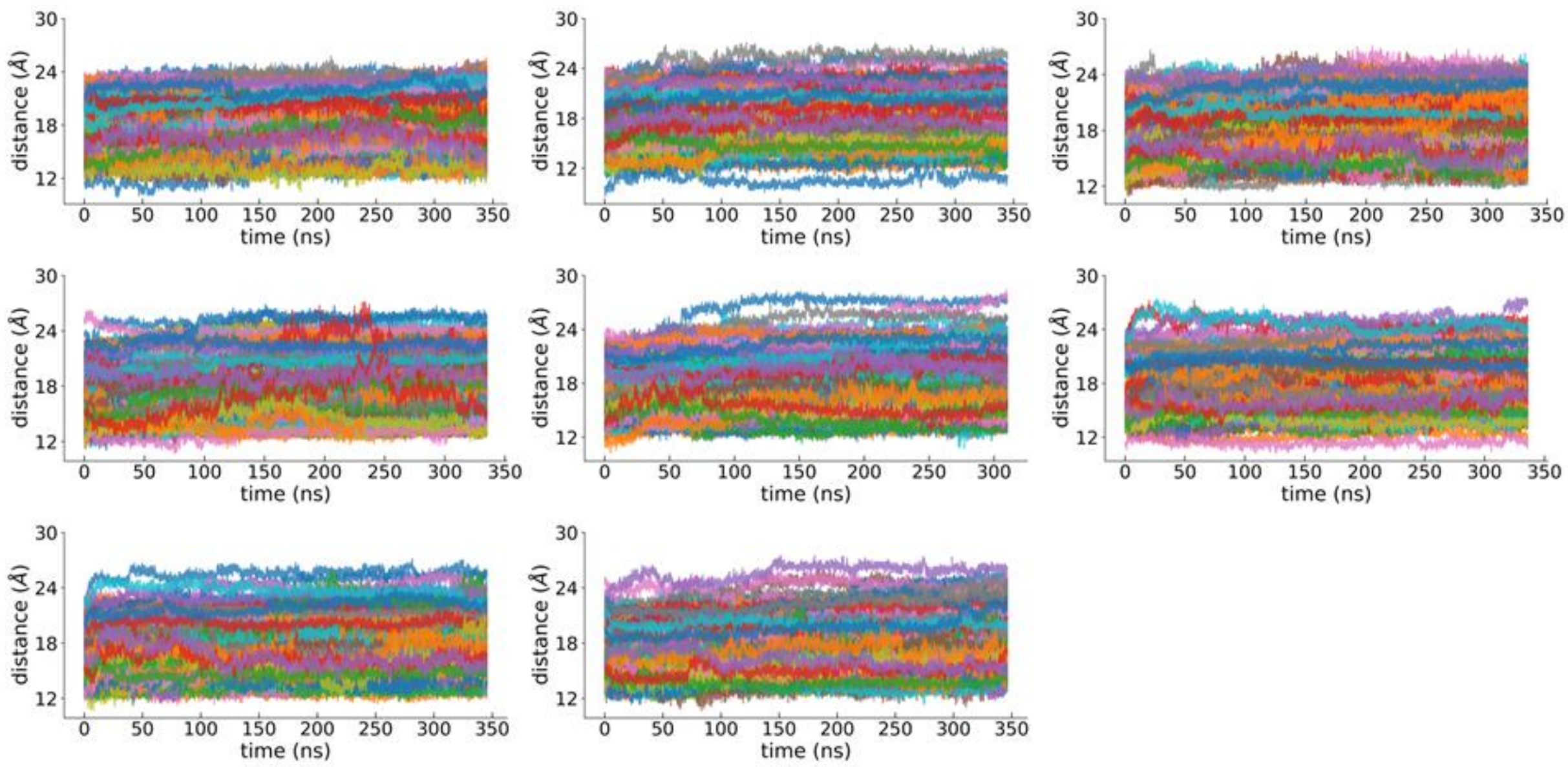

Figure S21 Structural variability of $PArg_{100}$ helices in the $PArg_{100}/PAsp_{50}$ square model during MD simulations. a) **Cα-Cα** distances (sampled for every tenth residue) between equivalent residues in adjacent $PArg_{100}$ chains. Each plotted line refers to a given **Cα-Cα** distance sampled in one MD simulation replica.

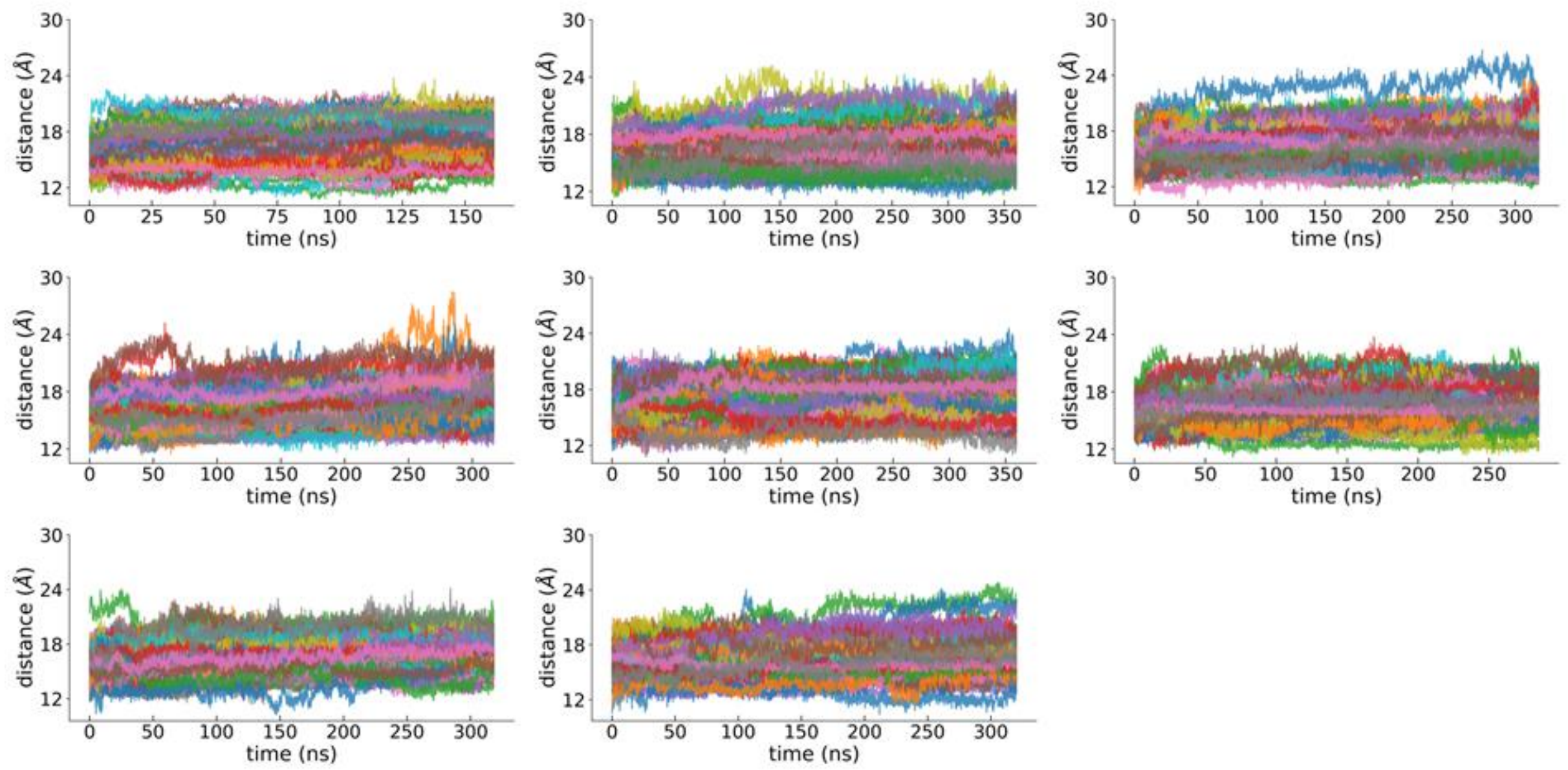

Figure S22 Structural variability of $PArg_{100}$ helices in the $PArg_{100}/PGlu_{50}$ square model during MD simulations. a) **Cα-Cα** distances (sampled for every tenth residue) between equivalent residues in adjacent $PArg_{100}$ chains. Each plotted line refers to a given **Cα-Cα** distance sampled in one MD simulation replica.

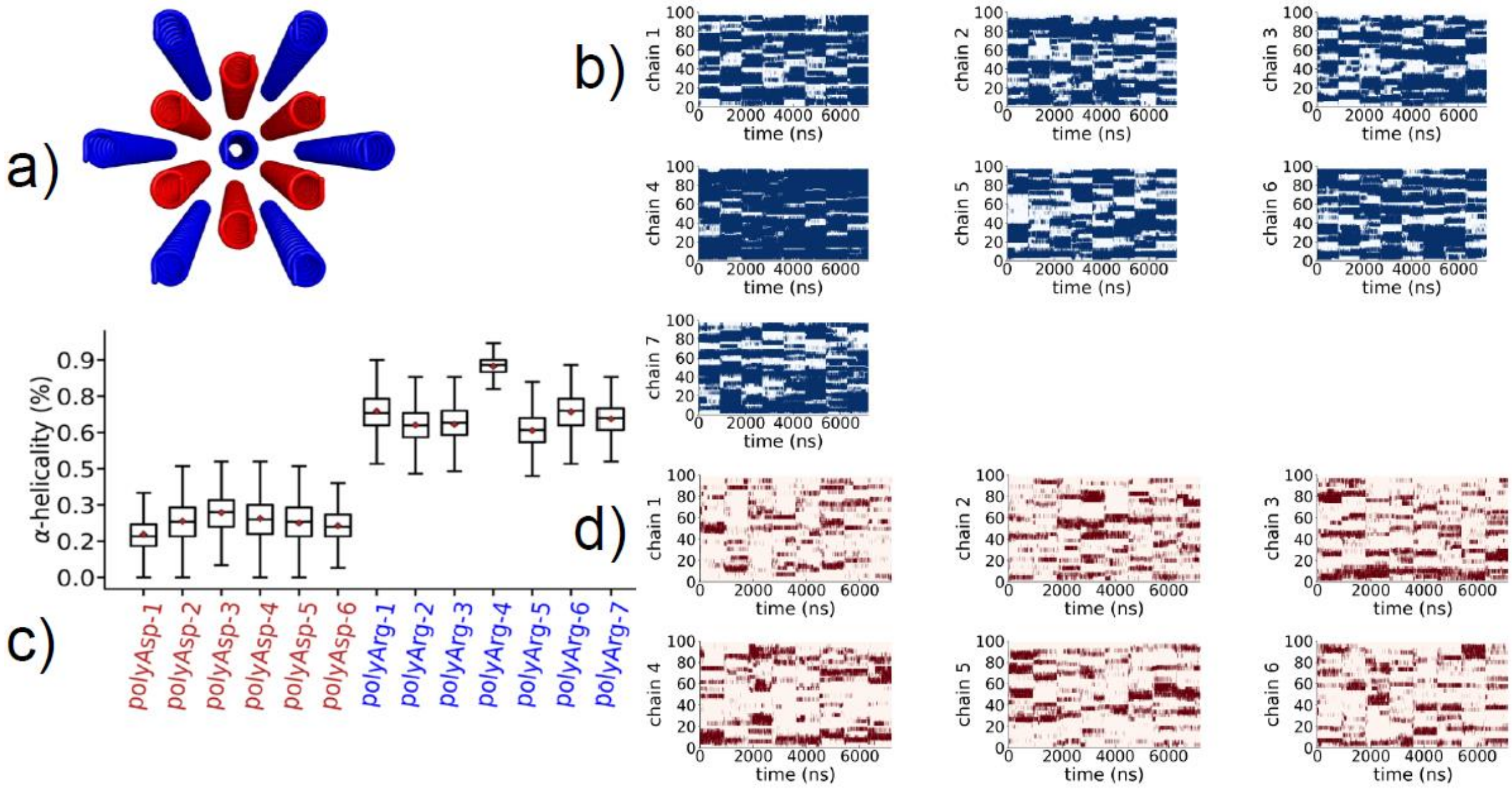

Figure S23 Structural dynamics of $PAsp_{100}$ chains in the $PArg_{100}/PAsp_{100}$ α-helical model. a) $PArg_{100}/PAsp_{100}$ model built with MODELLER. b) α-helical content of individual $PArg_{100}$ chains during MD simulations determined by DSSP (dark blue: α-helical, light blue: random coil). c) Proportion of residues adopting an α-helical structure in each $PArg_{100}$ chain (polyArg) and $PAsp_{100}$ chain (polyAsp) during MD simulations determined by DSSP. d) α-helical content of individual $PAsp_{100}$ chains during MD simulations determined by DSSP (dark red: α-helical, light red: random coil). In b) and d), each plot was generated by concatenating the results obtained for 8 simulation replicas end-to-end. The box plot rectangle indicates the interquartile range between the first and third quartiles, the whiskers delimitate the minimum and maximum value (excluding outliers), the red diamond and the horizontal line indicate the mean and median value, respectively.

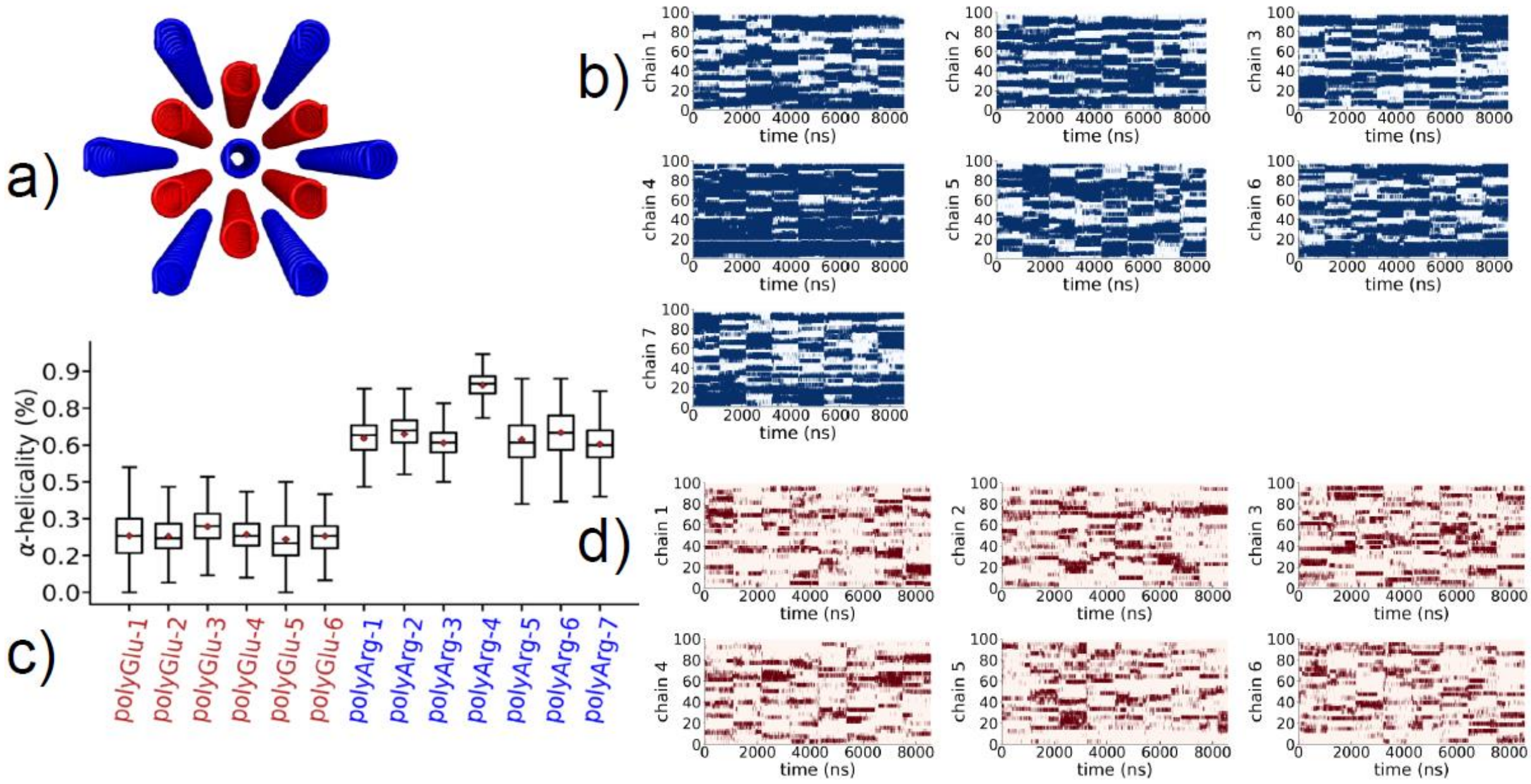

Figure S24 Structural dynamics of $PGlu_{100}$ chains in the $PArg_{100}$/$PGlu_{100}$ α-helical model. a) $PArg_{100}$/$PGlu_{100}$ model built with MODELLER. b) α-helical content of individual $PArg_{100}$ chains during MD simulations determined by DSSP (dark blue: α-helical, light blue: random coil). c) Proportion of residues adopting an α-helical structure in each $PArg_{100}$ chain (polyArg) and $PGlu_{100}$ chain (polyGlu) during MD simulations determined by DSSP. d) α-helical content of individual $PGlu_{100}$ chains during MD simulations determined by DSSP (dark red: α-helical, light red: random coil). In b) and d), each plot was generated by concatenating the results obtained for 8 simulation replicas end-to-end. The box plot rectangle indicates the interquartile range between the first and third quartiles, the whiskers delimitate the minimum and maximum value (excluding outliers), the red diamond, and the horizontal line indicates the mean and median value, respectively.

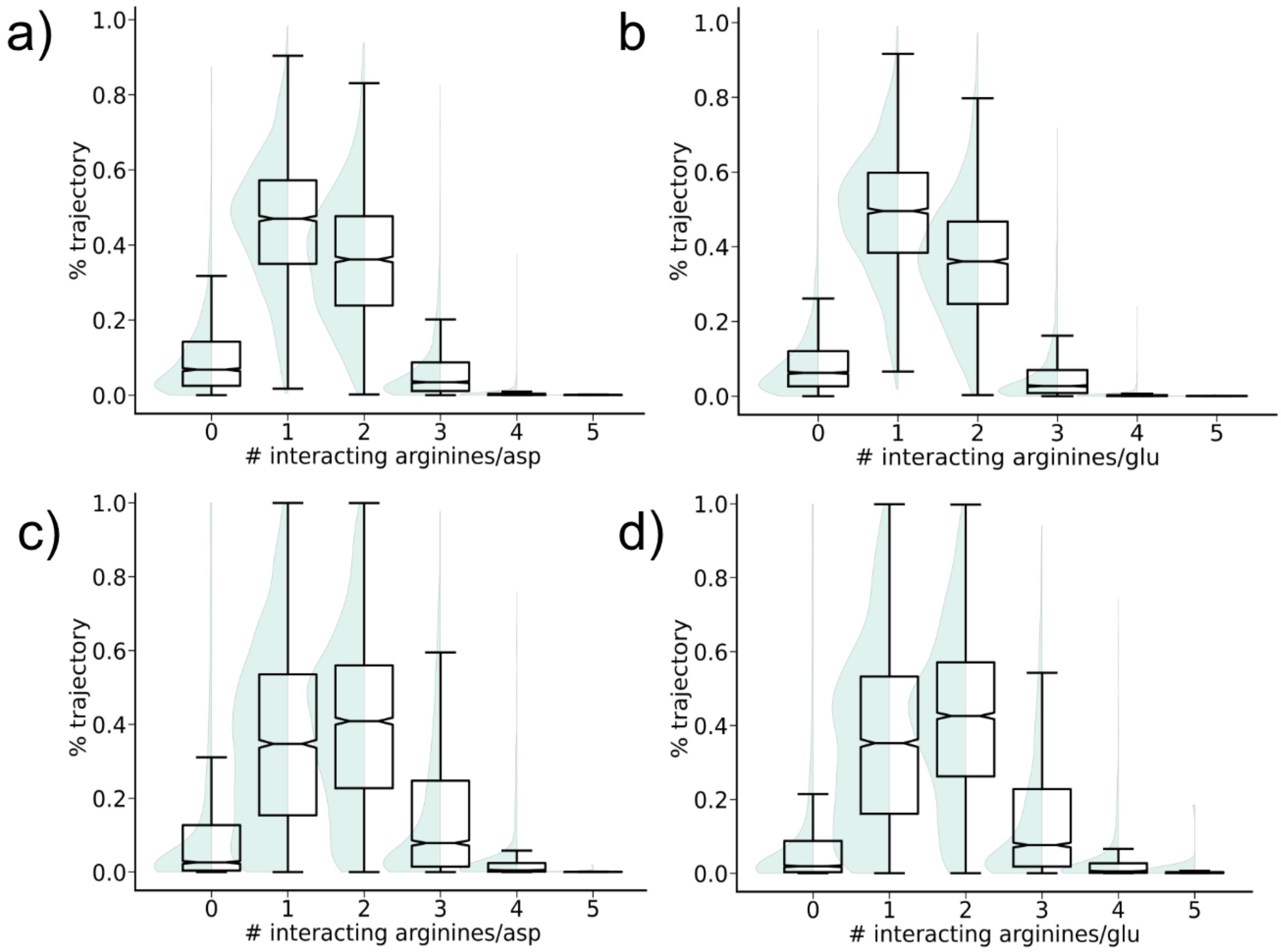

Figure S25 Occurrence of various types of salt-bridge interactions sampled in MD simulations. Hexagonal assemblies: a) $PArg_{100}/PAsp_{50}$ model, b) $PArg_{100}/PGlu_{50}$ model. Square assemblies: c) $PArg_{100}/PAsp_{50}$ model, d) $PArg_{100}/PGlu_{50}$ model. Cyan surface: kernel density estimations. The x-axes indicate the number of interacting arginine moieties per aspartate or glutamate residue.

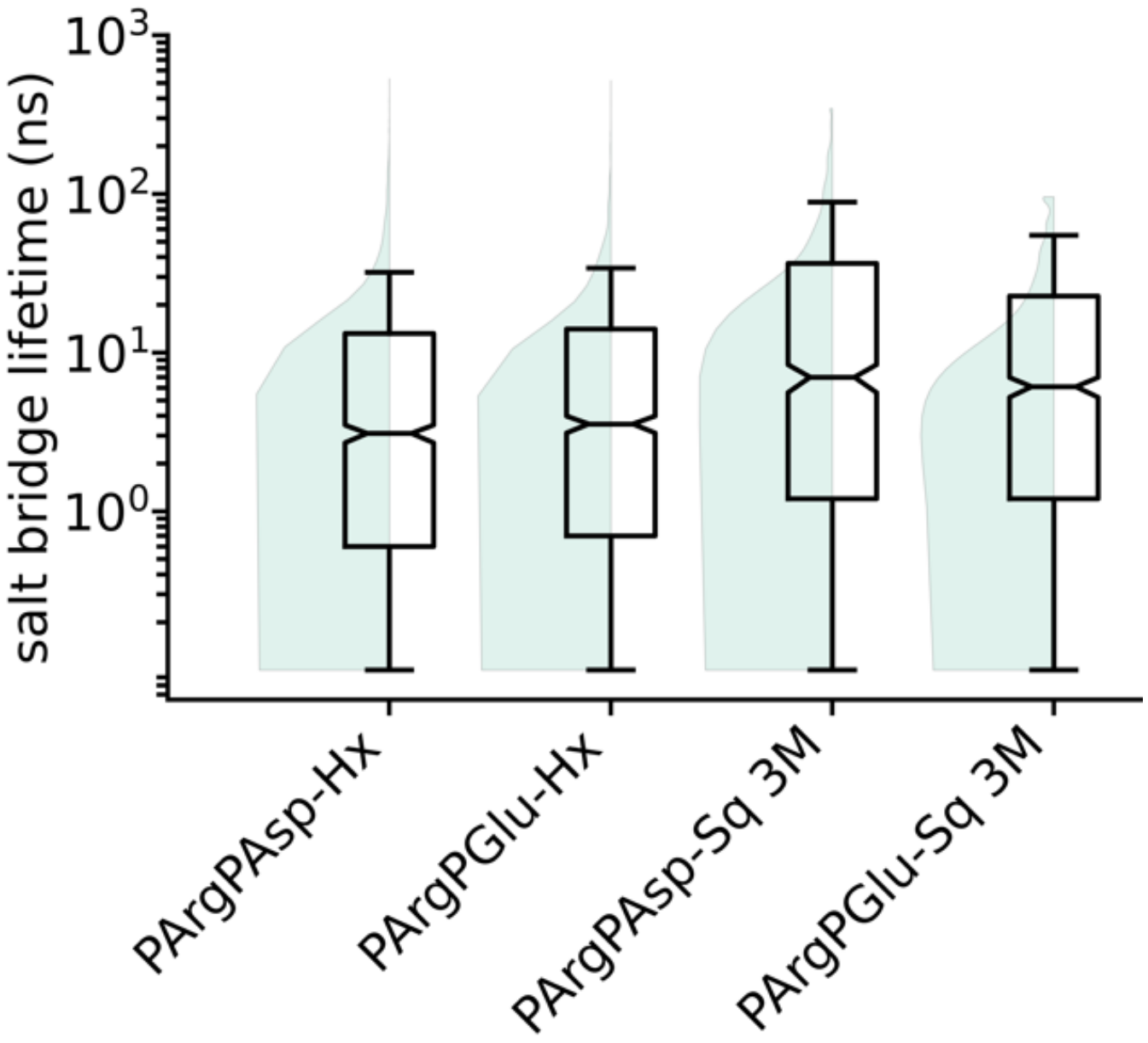

Figure S26 Maximum lifetimes distribution for all detected salt bridges in MD simulations. Hx: hexagonal assemblies. Sq 3M: square assemblies simulated in 3 M NaCl. Cyan surface: kernel density estimations.

7. NMR-investigations of phase separated complexes

a)

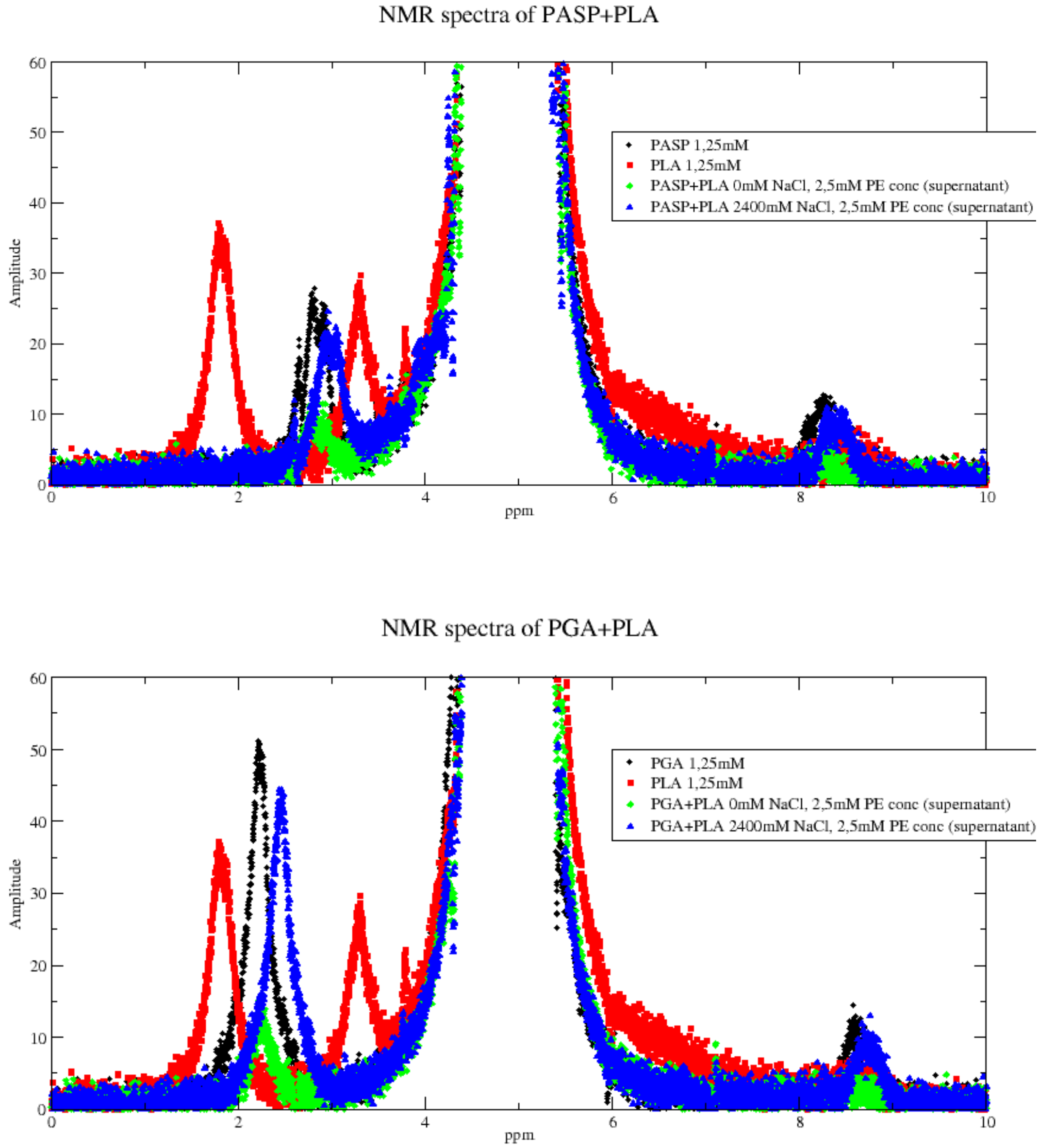

Figure S27. ^{1}H-NMR-spectra of the supernatants after complex formation for $PArg_{100}/PAsp_{100}$ (a) and $PArg_{100}/PGlu_{100}$. The spectra of pure PArg (red), PAsp (a, black) and PGlu (b, black) at 1.25 mM are shown as a reference. At no added salt (0 mM NaCl) we observe that some amount of the polyanions (green, a: PAsp, b: PGlu) remains in the supernatant. For high added salt (2400 mM) most of the polyanions (blue, a: PAsp, b: PGlu) remain in the supernatant. No free PArg is observed in the supernatant.

8. ^{13}C ssNMR of polyaminoacid conformation

We investigated the conformation of the PArg- and PAsp-chains by ^{13}C solid-state NMR (Fig. S28). For both arginine and aspartate the signals of C_α and C_β are rather sharp with line widths below 1.5 ppm, indicative of a high degree of homogeneity for both homopolymers. The C_α-signal of arginine at 60 ppm shows a secondary chemical shift of +3 ppm with respect to the random coil value of 57 ppm, which is indicative of α-helical conformation. The C_α-signal of the aspartate groups also has a secondary chemical shift of 3 ppm, which suggests that also a fraction of polyaspartate adopts helical conformation.

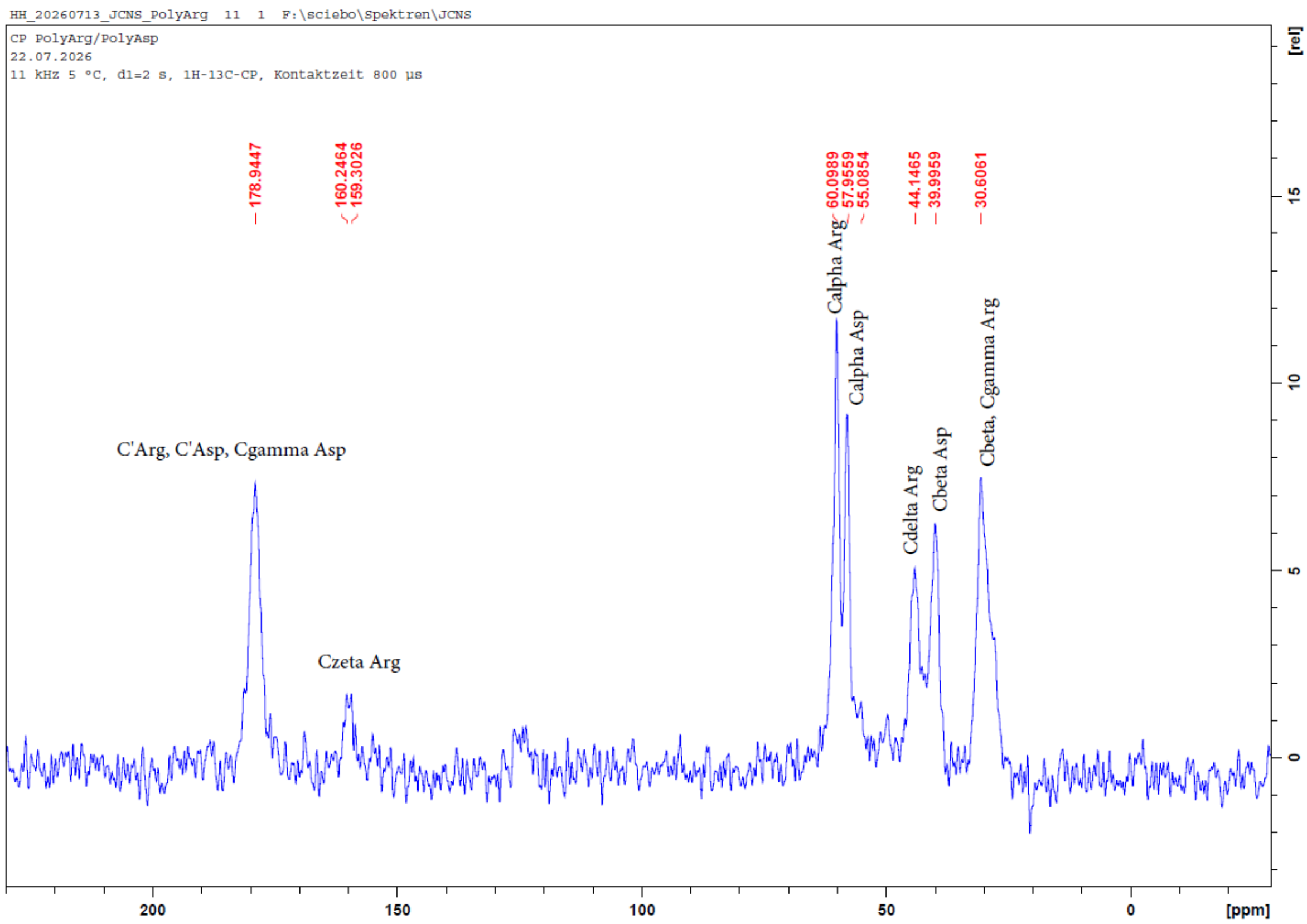

Figure S28. ^{13}C CP/MAS solid state NMR spectrum of the $PArg_{100}/PGlu_{100}$-complex prepared without additional salt. The three signals of Arg C', Asp C' and Asp Cg overlap to one characteristic carbonyl signal at 179 ppm. As indicated, we further observe the Arg C_ζ signal at 160 ppm, the Arg C_δ-signal at 44 ppm, the Asp C_β-signal at 40 ppm, and the Arg C_β and $C\gamma$ signals at 31 ppm. The sharp (half width of 1.25 ppm) signal of the Arg C_α-signal at 60 ppm indicates an α-helical conformation. The Asp C_α-signal at 58 ppm indicates a similar secondary structure. Sample spinning frequency was 11 kHz, the actual sample temperature was set to ~ 5 °C. The spectrum was processed with exponential multiplication with 50 Hz line broadening and referenced to DSS using adamantane as external standard.
